# E3 ligase HECTD3 promotes RNA virus replication and virus-induced inflammation via K33-linked polyubiquitination of PKR

**DOI:** 10.1038/s41419-023-05923-9

**Published:** 2023-07-04

**Authors:** Jiaying Huang, Zhou Yu, Xuelian Li, Mingjin Yang, Qian Fang, Zheng Li, Chunmei Wang, Taoyong Chen, Xuetao Cao

**Affiliations:** 1 Institute of Immunology, Zhejiang University School of Medicine, Hangzhou, 310058 China; 2 National Key Laboratory of Immunity and Inflammation, Suzhou Institute of Systems Medicine, Chinese Academy of Medical Sciences & Peking Union Medical College, Suzhou, 215123 Jiangsu China; 3 National Key Laboratory of Immunity and Inflammation & Institute of Immunology, Navy Medical University, Shanghai, 200433 China; 4 Institute of Immunology, College of Life Science, Nankai University, Tianjin, 300071 China

**Keywords:** Acute inflammation, Innate immunity

## Abstract

Uncontrolled viral replication and excessive inflammation are the main causes of death in the host infected with virus. Hence inhibition of intracellular viral replication and production of innate cytokines, which are the key strategies of hosts to fight virus infections, need to be finely tuned to eliminate viruses while avoid harmful inflammation. The E3 ligases in regulating virus replication and subsequent innate cytokines production remain to be fully characterized. Here we report that the deficiency of the E3 ubiquitin-protein ligase HECTD3 results in accelerated RNA virus clearance and reduced inflammatory response both in vitro and in vivo. Mechanistically, HECTD3 interacts with dsRNA-dependent protein kinase R (PKR) and mediates Lys33-linkage of PKR, which is the first non-proteolytic ubiquitin modification for PKR. This process disrupts the dimerization and phosphorylation of PKR and subsequent EIF2α activation, which results in the acceleration of virus replication, but promotes the formation of PKR-IKK complex and subsequent inflammatory response. The finding suggests HECTD3 is the potential therapeutic target for simultaneously restraining RNA virus replication and virus-induced inflammation once pharmacologically inhibited.

## Introduction

Upon infection by RNA viruses, the host elicits innate anti-viral response to eliminate viruses and then resolve viral infection-induced inflammation for maintaining homeostasis [1]. After recognizing viral RNA that is delivered to the cytosol in the form of incoming viral genomes or generated through viral RNA replication [2], the host can eliminate viruses through three processes. Rig-I like receptors (RLRs) recognize virus-derived pathogenic RNA in the cytoplasm, activate downstream signaling, and ultimately induce type I interferon and inflammatory factor production to elicit anti-viral response [3]. Oligoadenylate synthetases (OASs) recognize viral-derived RNA and promote the dimerization and activation of RNAase L to degrade viral RNA and thus inhibit viral replication [4]. Protein Kinase R (PKR) recognizes viral-derived RNA and promotes its own dimerization and autophosphorylation, leading to the activation of Eukaryotic translation Initiation Factor 2α (EIF2α)-mediated protein translation inhibition and cell apoptosis, ultimately restricting intracellular replication of virus [5]. Eliminating viral replication and avoiding excessive inflammation through the synergistic action of the above mechanisms is the optimal outcome of antiviral immune response while its internal regulatory mechanisms are still needed further elucidated.

PKR is a protein of 551 amino acids that contains a C-terminal kinase domain and two N-terminal double-stranded RNA-binding motifs (dsRBMs) [6]. The dsRNA molecules are recognized by PKR through dsRBMs and resulting in PKR dimerization and autophosphorylation that represents its activation [5]. In addition to inhibiting protein translation by phosphorylation of EIF2α, PKR’s activation also regulates inflammation, cell proliferation, and cell death through NF-κB, P53, and FADD/caspase8 signals, thus playing critical role in homeostasis regulation [7–11]. Nevertheless, how PKR’s activation is regulated by post-translational modification especially ubiquitination is still unclear and requires further elucidate.

The ubiquitin (Ub) modification of proteins plays a key role in the regulation of immune response [12]. E3 ligases mediate the ubiquitination of substrate proteins, resulting in changing functions of these proteins [13]. Several E3s have been reported to play crucial roles in innate anti-viral responses but their importance in the anti-viral signaling still needs to be further studied [14, 15]. For example, HECTD3 has been reported to catalyze the K63-linked polyubiquitination of TRAF3, resulting in promoting the TRIF/STING-mediated innate anti-bacterial and anti-pathogenic DNA immune responses [16]. However, it remains unclear if HECTD3 has a role in anti-pathogenic RNA response or if HECTD3 mediates other than K63-linked polyubiquitination and ubiquitination of substrates other than TRAF3 in innate immune response.

Several key E3 ubiquitin ligases have been identified by us to be involved in the regulation of anti-viral immune response [17–19]. Our preliminary observation suggested that the mRNA expression of the E3 ligase HECTD3 was downregulated in RAW264.7 cells with vesicular stomatitis virus (VSV) infection (GEO accession number GSE72077). In this study, we provide in vitro and in vivo evidence demonstrating critical role of HECTD3 in promoting RNA virus replication and virus-induced inflammation. We reveal that HECTD3 binds to PKR and catalyzes K33-linked polyubiquitination of PKR, which inhibits PKR dimerization and phosphorylation that deprive it of restraining viral replication. On the other hand, K33-linked PKR prefers to form complex with IKK signalosome and activate downstream innate inflammatory signal. Our findings add mechanistic insight to the understanding of HECTD3 in promotion of viral replication and virus-induced inflammation, and also propose a potential target to be pharmacologically inhibited for suppressing viral infection and innate inflammation.

## Results

### Deficiency of HECTD3 simultaneously inhibits RNA virus replication and virus-induced cytokine production in vitro

We found that the mRNA and protein levels of HECTD3 were downregulated in mouse peritoneal macrophages after VSV infection (Supplementary Fig. 1a, b). Then we investigated the underlying mechanism and found that the protein level of HECTD3 also had a reduction in the presence of Actinomycin D (Act D, a transcription inhibitor) and this process was not affected by VSV infection (Supplementary Fig. 1c). When MG132 was used to inhibit proteasome-mediated protein degradation, the amount of HECTD3 increased gradually while this process was reversed by VSV infection (Supplementary Fig. 1d). These results suggested that VSV mainly inhibited the expression of HECTD3 at transcriptional level rather than proteasome-mediated degradation. Functionally, we found that *Hectd3* knockdown (KD) (Supplementary Fig. 1e) inhibited both mRNA and protein levels of IL-6 and IFNβ induced by VSV infection (Supplementary Fig. 1f–i).

**Fig. 1:**
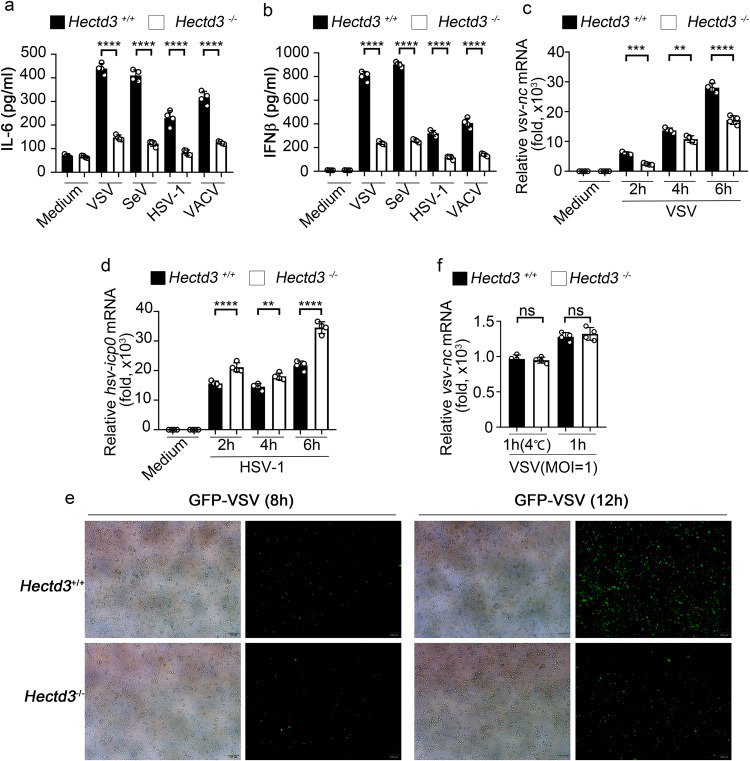
HECTD3 deficiency impairs RNA virus-induced innate cytokine production and restrains virus replication. **a**, **b** *Hectd3*^*+/+*^ or *Hectd3*^*–/–*^ BMDM (2 × 10^5^ cells per 24-well) were stimulated with the indicated pathogens for 6 h (MOI = 1 for VSV and SeV and MOI = 5 for HSV-1 and VACV). The amount of IL-6 (**a**) and IFNβ (**b**) in supernatants were measured by ELISA. **c**, **d** *Hectd3*^*+/+*^ or *Hectd3*^*–/–*^ BMDM (2×10^5^ cells per 24-well) were infected with VSV (MOI = 1) (**c**) or HSV-1 (MOI = 5) (**d**) for 2 h, 4 h, 6 h. The mRNA level of VSV nucleocapsid (nc) (**c**) or HSV-1 ICP0 (**d**) gene was examined by Q-PCR. **e** *Hectd3*^*+/+*^ or *Hectd3*^*–/–*^ BMDM (2 × 10^5^ cells per 24-well) were infected with GFP-VSV (MOI = 1) virus for 12 h, then examined by fluorescence microscope (Scale bar, 100 µm). **f** *Hectd3*^*+/+*^ or *Hectd3*^*–/–*^ BMDM (2×10^5^ cells per 24-well) were infected with VSV (MOI = 1) virus and incubated at 4 °C or 37 °C for 1 h as indicated. The mRNA level of VSV nucleocapsid (nc) gene was examined by Q-PCR. Results are presented as mean ± SD of three biological replicates (**a**–**d**, **f**) (one-way ANOVA followed by Bonferroni multiple comparison). One representative experiment of three is shown. ns, no significance; **P < 0.01; ****P < 0.0001.

To further investigate the role of HECTD3 in the innate anti-viral immune response, we established *Hectd3* knockout mice. The mice could reproduce normally and conform to Mendel’s laws of inheritance. The appearance, growth and life span of *Hectd3*^*−/−*^ mice were normal and the distribution of immune cells in spleen and lymph nodes (LN) was not significantly different (Supplementary Fig. 2). When *Hectd3*^*−/−*^ BMDM were infected with RNA viruses (VSV and Sendai virus–SeV) and DNA virus (HSV-1 and VACV), the production of IL-6 and IFNβ was significantly reduced (Fig. 1a, b). Unexpectedly, the replication of VSV was also inhibited in *Hectd3*^*−/−*^ BMDM (Fig. 1c), while the replication of HSV-1 was enhanced in *Hectd3*^*−/−*^ BMDM which consistent with common sense (Fig. 1d). When BMDM was infected with GFP-tagged VSV, the fluorescence intensity of wild-type BMDM cells was also significantly higher than that of *Hectd3*^*−/−*^ BMDM (Fig. 1e). These data suggested that HECTD3 played different roles, inhibiting or promoting, in anti-RNA virus and anti-DNA virus replication responses. By measuring the viral load on the cell surface or inside the cell at the early stage of infection, we found that HECTD3 did not affect the amount of the virus to adhere to or enter into macrophages (Fig. 1f). Furthermore, we validated the anti-RNA virus effect in *Hectd3*^*−/−*^ L929 fibroblast cell (Supplementary Fig. 3a–d) and also found that knockdown HECTD3 inhibited IL-6 and IFNβ production (Supplementary Fig. 4a–c), and restrained virus replication (Supplementary Fig. 4d) in human monocyte-macrophage THP-1 cells once infected with GFP-VSV. Therefore, we hypothesized that HECTD3 selectively promotes RNA viral replication and subsequent virus-induced inflammatory innate responses.

### Deficiency of HECTD3 restrains RNA virus propagation and virus-induced inflammation in vivo

**Fig. 2:**
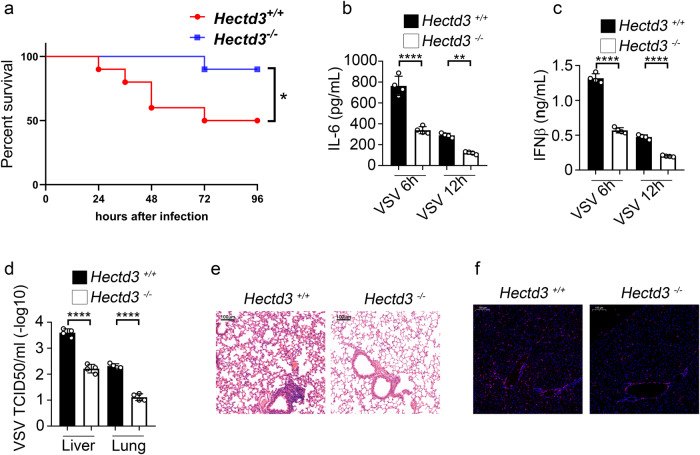
HECTD3 deficiency impairs innate response and restricts virus propagation in vivo. **a** *Hectd3*^*+/+*^ or *Hectd3*^*–/–*^ mice were intraperitoneally injected with 2 × 10^7^ PFUs of VSV. The survival of mice (*n* = 10 per group) was monitored by Kaplan and Meier method and analyzed by Log-rank test. **b**, **c** *Hectd3*^*+/+*^ or *Hectd3*^*–/–*^ mice were intraperitoneally injected with 5 × 10^6^ PFUs of VSV and their serum levels of IL-6 (**b**) and IFNβ (**c**) were measured by ELISA 6 h or 12 h after VSV infections as indicated (*n* = 4 mice per group). **d** 24 h after VSV infections, viral titers in the homogenate supernatant of indicated organs from *Hectd3*^*+/+*^ or *Hectd3*^*–/–*^ mice were determined by TCID50 assay. **e** H&E staining of lung sections from mice (in **e**). Scale bars, 100 µm. **f** Immunofluorescent staining for CD45 in liver sections from mice (in **d**). Scale bars, 100 µm. Results are presented as mean ± SD (**b**–**d**) (one-way ANOVA followed by Bonferroni multiple comparison). One representative experiment of three is shown. *P < 0.05; **P < 0.01; ****P < 0.0001.

We further studied the in vivo effects of HECTD3. After intraperitoneal injection of VSV, we found that *Hectd3*^*−/−*^ mice survived significantly longer than *Hectd3*^+/+^ mice (Fig. 2a) and that serum levels of IL-6 and IFNβ as well as virus titers in the lung and liver of *Hectd3*^*−/−*^ mice were significantly lower than those of *Hectd3*^+/+^ mice (Fig. 2b–d). Furthermore, we observed less severe pathological damages in the lung (Fig. 2e) and fewer infiltration of CD45^+^ cells in the liver of *Hectd3*^*−/−*^ mice than *Hectd3*^+/+^ mice (Fig. 2f). These data showed that HECTD3 deficiency protected mice from the RNA virus replication and virus-induced inflammation.

### HECTD3 interacts with PKR and suppresses RNA virus-triggered PKR-EIF2α and apoptosis pathway

**Fig. 3:**
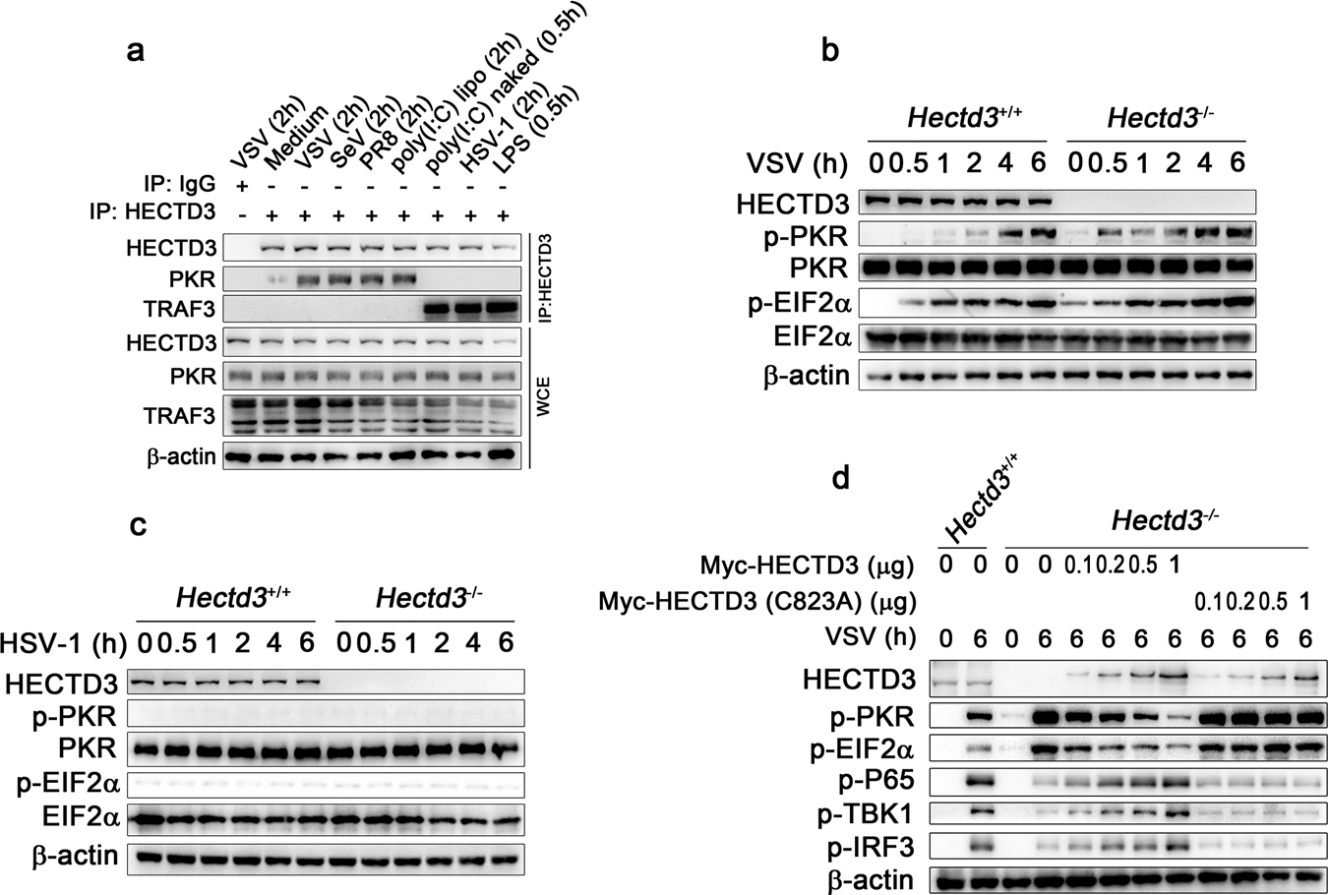
HECTD3 interacts with PKR and impairs the activation of PKR-EIF2α pathway. **a** Wild-type BMDM were infected with VSV (MOI = 1), SeV (MOI = 1), PR8 (MOI = 1), HSV-1 (MOI = 5) for 2 h or stimulated with liposome-packaged poly (I:C) (1 µg/ml), naked poly (I:C) (5 µg/ml), or LPS (100 ng/ml) for 0.5 h or 2 h as indicated. Whole-cell extracts (WCE) were immunoprecipitated with anti-HECTD3 antibody plus protein A/G beads then immunoblotted to detected indicated proteins. **b**, **c** *Hectd3*^*+/+*^ or *Hectd3*^*–/–*^ BMDM were infected with VSV (MOI = 1) (**b**) or HSV-1 (MOI = 5) (**c**) for 0 h, 0.5 h, 1 h, 2 h, 4 h, 6 h as indicated and the activation of PKR-EIF2α signaling pathway were examined by Western blotting. **d** *Hectd3*^*+/+*^ or *Hectd3*^*–/–*^ RAW264.7 cells with reconstitution of Myc-HECTD3 or Myc-HECTD3(C823A) were infected with VSV (MOI = 1) for 0 h or 6 h as indicated and the activation of the signaling mediators were examined by Western blotting.

HECTD3 is an E3 ubiquitin ligase performing its function mainly by binding to and modifying substrate proteins through ubiquitination. To explore the mechanism of HECTD3 in promoting viral replication and subsequent inflammatory process, we therefore went on to identify binding partners of HECTD3 by using immunoprecipitations (IP) after overexpression of Myc-tagged HECTD3 in HEK293T cells followed by mass spectrometry. Among the potential HECTD3 binding proteins identified, we found PKR as the candidate which was reported to combat RNA virus infection by inhibiting viral protein translation as mentioned before (Supplementary Fig. 5). We confirmed the interaction between the endogenous HECTD3 and the endogenous PKR in BMDM, and found the interaction was enhanced by RNA viruses and cytosolic RNA analogues but not by the DNA virus HSV-1 or TLR ligands (naked poly I:C and LPS) (Fig. 3a). We didn’t observe the interaction between HECTD3 and TRAF3 in anti-RNA responses which exists in the anti-DNA virus response and TLR signal (Fig. 3a). Functionally, we found that HECTD3 deficiency increased the phosphorylation of PKR and EIF2α in BMDM infected with VSV (Fig. 3b) but not HSV-1 (Fig. 3c). For another, HECTD3 deficiency decreased the phosphorylation of IKKβ, P65, TBK1, and IRF3 in BMDM infected with VSV (Supplementary Fig. 6a), but affected only the activation of TBK1-IRF3 in macrophages infected by HSV-1 (Supplementary Fig. 6b). Furthermore, reconstitution of HECTD3, but not HECTD3 (C823A) mutant (lacks E3 ligase activity), in *Hectd3*^*−/−*^ RAW264.7 cells dose-dependently decreased VSV infection-induced phosphorylation of PKR and EIF2α and promoted the activation of NF-κB and TBK1-IRF3 signalings (Fig. 3d). PKR was reported to mediate Caspase8 dependent cell apoptosis [11], but we didn’t find significant apoptosis of BMDMs at the early stage of viral infection (12 h) (data not shown). Our result showed that *Hectd3*^*−/−*^ BMDM were more susceptive to VSV-induced apoptosis than wild-type BMDM cells after infected for 24 h (Supplementary Fig. 7a) and VSV could induce more cleavage of Caspase8 and Caspase3 in *Hectd3*^*−/−*^ BMDMs as compared to wild-type cells (Supplementary Fig. 7b). These data suggested that HECTD3 inhibited the PKR-EIF2α signaling which is responsible for restricting RNA virus replication, and enhanced the innate signaling for inflammatory cytokine production which probably due to the increased viral load in the early stage of RNA virus infection. On the other hand, HECTD3-PKR mediated cell apoptosis may affect the propagation of RNA virus in the later stages of infection.

**Fig. 4:**
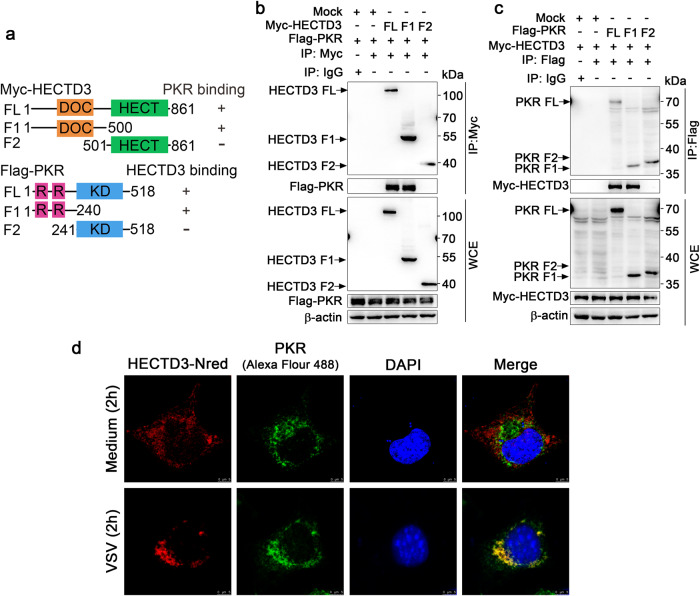
The DOC domain of HECTD3 binds the RNA-binding domain of PKR. **a** Schematic illustration of domains in the full-length (FL) or fragments (F) of Myc-HECTD3 or Flag-PKR. DOC, DOC domain; HECT, HECT domain; R, RNA binding domain; KD, kinase domain. Numerical numbers indicate for the site of amino acids. **b**, **c** HEK293T cells were transiently transfected with plasmids expressing Flag-tagged wild-type PKR or PKR mutants and Myc-tagged wile type HECTD3 or HECTD3 mutants as indicated for 48 h. Whole-cell extracts (WCE) were immunoprecipitated with anti-Myc Sepharose Beads (**b**) or anti-Flag Sepharose Beads (**c**) then immunoblotted for indicated proteins. **d** RAW264.7 cells grown on cover slides were transiently transfected with a plasmid expressing N-terminal red fluorescent protein (Nred)-tagged HECTD3 for 48 h, and then infected with VSV (MOI = 1) viruses for 2 h. After counterstained with PKR (Alexa Flour 488) and DAPI, cells were examined by confocal microscope (Scale bar, 5 µm). One representative experiment of three is shown.

After coexpression of Myc-Tagged wild-type HECTD3 or HECTD3 mutants with Flag-tagged wild-type PKR or PKR mutants (Fig. 4a), we found that the N-terminal DOC domain of HECTD3 could interact with the N-terminal RNA-binding domain of PKR (Fig. 4b, c). Furthermore, ectopically expressed HECTD3 partially colocalized with the endogenous PKR in the cytoplasm of RAW264.7 macrophages and the colocalization was enhanced by VSV infection (Fig. 4d). Therefore, PKR might be the modified and regulated substrate for HECTD3 in the innate response to infection of RNA virus but not DNA virus.

### HECTD3 mediates Lys33-linked polyubiquitination of PKR

**Fig. 5:**
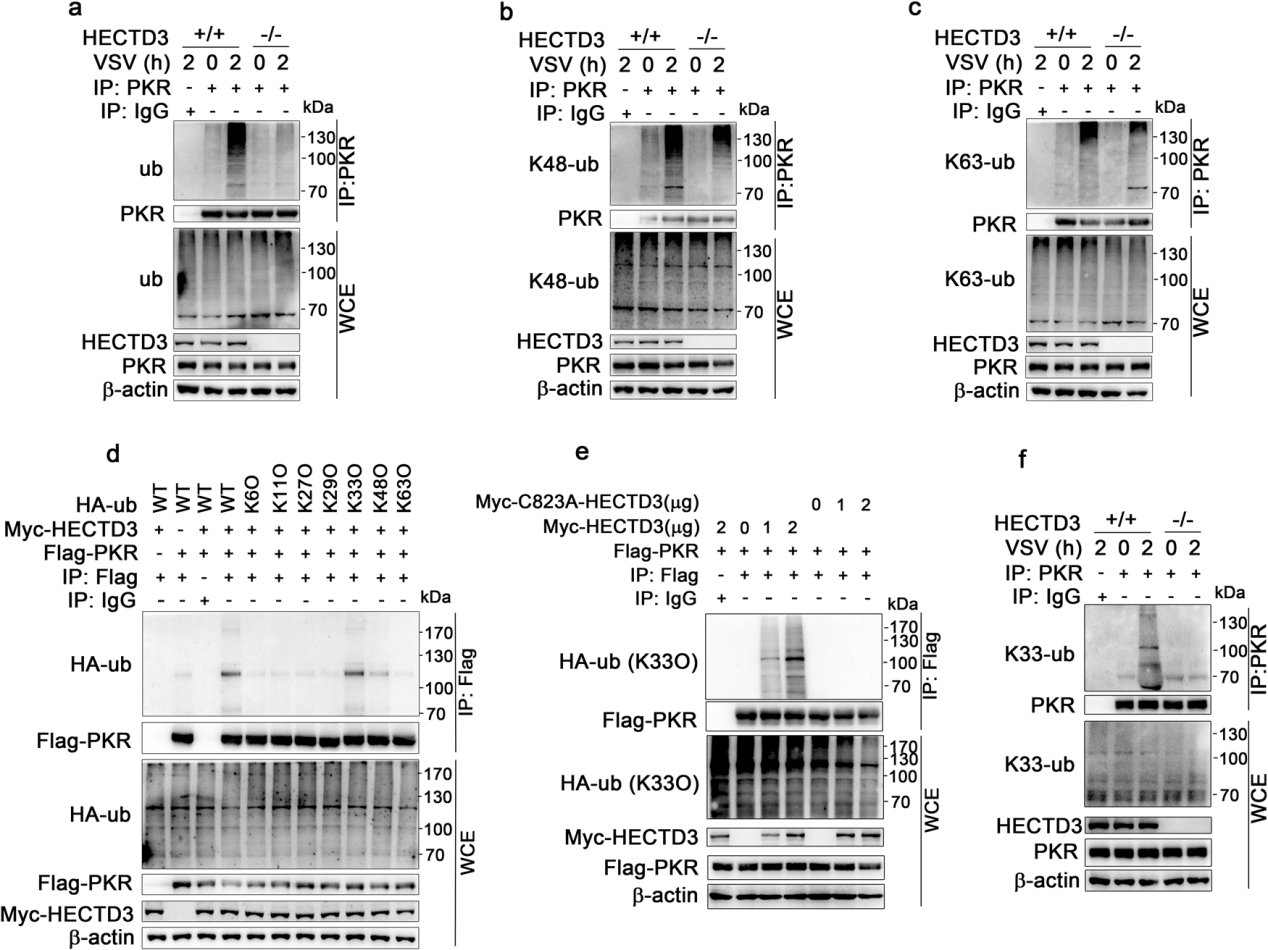
HECTD3 mediates K33-linked polyubiquitination of PKR during RNA virus infection. **a**–**c**, **f** *Hectd3*^*+/+*^ or *Hectd3*^*–/–*^ BMDM were infected with VSV (MOI = 1) for 2 h, whole-cell extracts (WCE) were heated in buffer containing 1% SDS then immunoprecipitated (IP) with anti-PKR antibody plus protein A/G beads. Polyubiquitination (wild-type) (**a**), K48-linked polyubiquitination (**b**), K63-linked polyubiquitination (**c**) and K33-linked polyubiquitination (**f**) of PKR were examined by Western blotting. **d** HEK293T cells were transiently transfected with plasmids expressing Myc-tagged HECTD3, Flag-tagged PKR and HA-tagged wild-type or mutant Ub as indicated for 48 h. Then polyubiquitination of PKR was examined by Western blotting after immunoprecipitations with anti-Flag Sepharose Beads. **e** HEK293T cells were transiently transfected with plasmids expressing HA-tagged K33O ub, Myc-tagged wild-type or mutant HECTD3 and Flag-tagged PKR as indicated for 48 h. Then polyubiquitination of PKR was examined by Western blotting against HA after immunoprecipitations with anti-Flag Sepharose Beads. One representative experiment of three is shown.

As an E3 ubiquitin ligase, HECTD3 may promote the ubiquitination of its substrate(s). As expected, we found that polyubiquitination of PKR was lower in *Hectd3*^*−/−*^ BMDM than that in *Hectd3*^+/+^ BMDM after VSV infection (Fig. 5a). Lys48 (K48)-linked and Lys63 (K63)-linked polyubiquitination are most common types of polyubiquitination. However, our results did not support HECTD3-mediated either K48- or K63-linked polyubiquitination of PKR (Fig. 5b, c).

To clarify the type of ubiquitination of PKR mediated by HECTD3, we cotransfected plasmids expressing HECTD3 and HA-tagged wild-type Ub or Ub mutant with only one lysine residue unchanged (K6O, K11O, K27O, K29O, K33O, K48O, and K63O). We found that HECTD3 mainly mediated K33-linked polyubiquitination of PKR (Fig. 5d). Consistently, when K33O mutant Ub was introduced into HEK293T cells, the wild-type HECTD3 but not the HECTD3 C823A mutant, which lacks the E3 ligase activity, dose dependently promoted the K33-linked polyubiquitination of PKR (Fig. 5e). In *Hectd3*^*−/−*^ BMDM, the K33-linked polyubiquitination of PKR was significantly decreased than that in *Hectd3*^+/+^ BMDM after VSV infection, which was detected by K33-linked polyubiquitin antibody (Fig. 5f).

We then went to identify the sites for K33-linked Ub chain in PKR. We constructed 37 mutants for PKR (No. 1-37, mutated indicated Lys residue(s) into Arg) which covering all 52 Lys sites in PKR, and examined the K33-linked polyubiquitination associated with PKR in HEK293T cells. We found that the No.6 mutant of PKR (mutation of Lys68 into Arg) was the only mutant that could not be modified by the K33 polyubiquitin chain effectively in the presence of HECTD3 (Supplementary Fig. 8). These data suggested that HECTD3 was an E3 ubiquitin ligase mediating K33-linked polyubiquitination of PKR and the Lys68 of PKR may be the major ubiquitination site modified by HECTD3 in the innate response to RNA virus infection.

### HECTD3 inhibits replication-restraining PKR dimerization and PKR-EIF2α phosphorylation through its E3 ligase activity

**Fig. 6:**
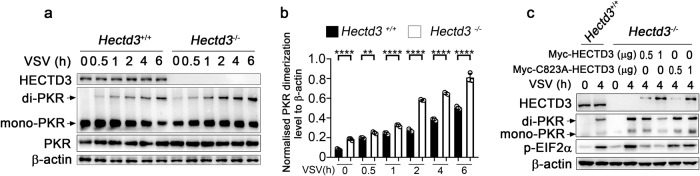
Polyubiquitination of PKR catalyzed by HECTD3 inhibits PKR dimerization. **a** The dimerization of PKR in *Hectd3*^+/+^ or *Hectd3*^–/–^ BMDM infected with VSV (MOI = 1) as indicated was examined by native page and Western blot. **b** Quantification analysis of western blotting results in (**a**). β-actin was used as a control. **c** The dimerization of PKR in *Hectd3*^+/+^ or *Hectd3*^–/–^ RAW264.7 cell with reconstitution of Myc-HECTD3 or Myc-HECTD3 (C823A) and infected with VSV (MOI = 1) for 0 h or 4 h as indicated was examined by native page and Western blot. Results are presented as mean ± SD of three biological replicates (**b**) (one-way ANOVA followed by Bonferroni multiple comparison). One representative experiment of three is shown. **P < 0.01; ****P < 0.0001.

After RNA virus infection, PKR can inhibit viral replication by dimerization and activating EIF2α. In the above study, we found that the Lys68 of PKR which ubiquitinated by HECTD3 is located in the dsRBM domain that mediates PKR dimerization [5]. We therefore hypothesized that HECTD3-mediated K33-linked polyubiquitination of PKR might regulate the formation of PKR dimer. Indeed, HECTD3 deficiency promoted the dimerization of PKR in macrophages during VSV infection (Fig. 6a, b). The ectopic expression of HECTD3 but not the HECTD3 (C823A) mutant in *Hectd3*^*−/−*^ RAW264.7 cells dose dependently reduced the VSV infection-induced dimerization of PKR and activation of EIF2α (Fig. 6c). These data suggested that the ubiquitin ligase activity of HECTD3 is required for the prevention of PKR dimerization which deprives PKR of the ability to suppress virus replication.

### PKR is required for HECTD3-mediated antiviral innate response

**Fig. 7:**
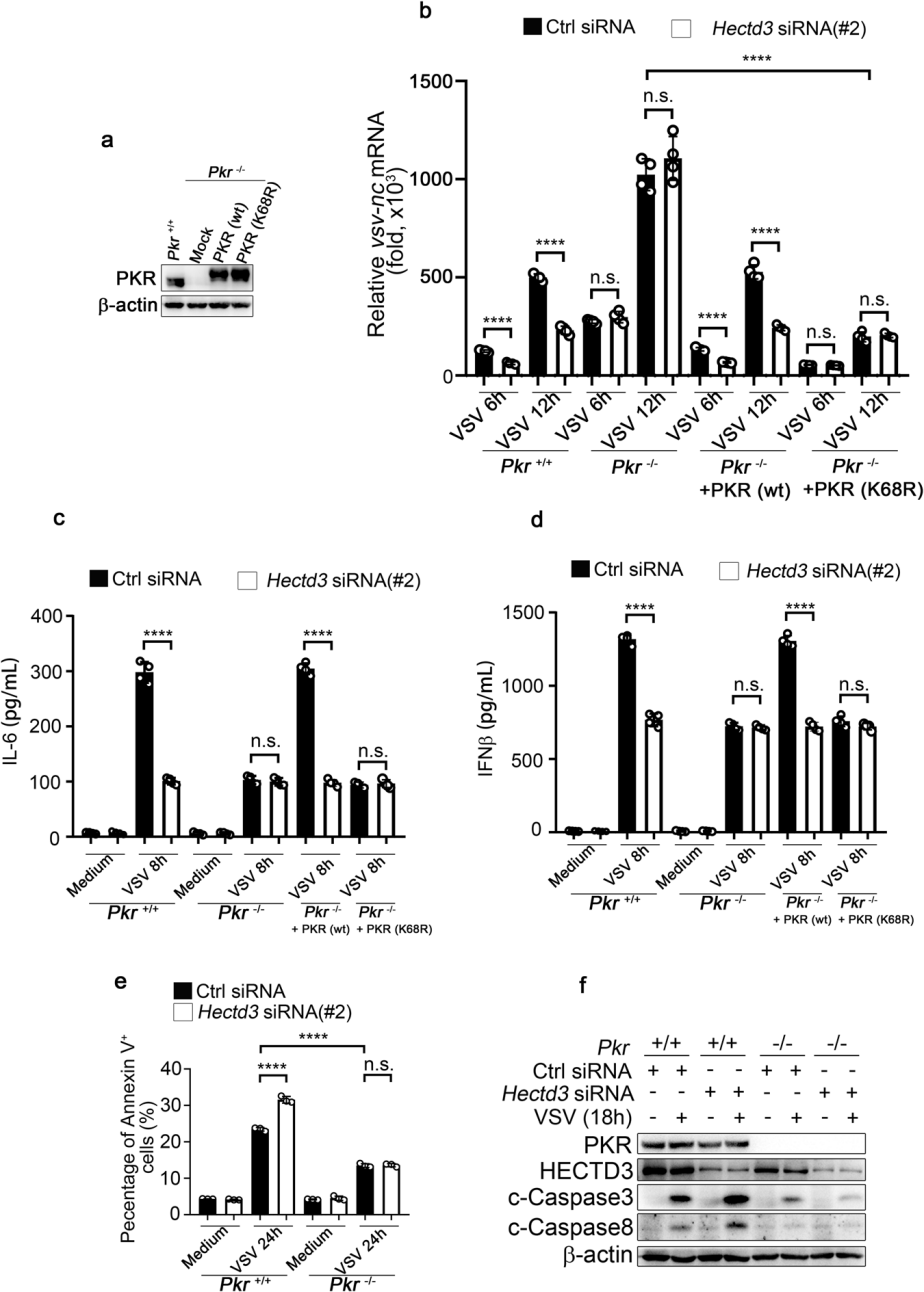
PKR is required for HECTD3-mediated anti-viral responses against RNA virus. **a** *Pkr*^–/–^ Raw264.7 cells were transfected with plasmids expressing Flag-tagged PKR (wt) or PKR (K68R). The efficiency of reconstitution was evaluated by Western blotting. **b** *Pkr*^+/+^ or *Pkr*^–/–^ Raw264.7 cells reconstituted with PKR (wt) or PKR (K68R) were transfected with control (Ctrl) or *Hectd3* siRNA (#2) for 48 h and infected with VSV (MOI = 1) as indicated. The mRNA level of VSV nucleocapsid (nc) was examined by Q-PCR. **c**, **d** *Pkr*^*+/+*^ or *Pkr*^*–/–*^ Raw264.7 cells reconstituted with PKR (wt) or PKR (K68R) were transfected with control (Crtl) or *Hectd3* siRNA(#2) as indicated and infected with VSV (MOI = 1) for 8 h. Amounts of IL-6 (**c**) and IFNβ (**d**) in supernatants were measured by ELISA. **e** Quantification of the percentage of ANNEXIN V^+^ *Pkr*^*+/+*^ or *Pkr*^*–/–*^ Raw264.7 cells which were transfected with control (Ctrl) or *Hectd3* siRNA (#2) as indicated and infected with VSV (MOI = 1) for 24 h as indicated time. Cells were washed by PBS, stained with ANNEXIN V-FITC for 15 min at RT, and subjected to cell apoptosis analysis by flow cytometry. **f** *Pkr*^*+/+*^ or *Pkr*^*–/–*^ Raw264.7 cells were transfected with control (Ctrl) or *Hectd3* siRNA (#2) as indicated and infected with VSV(MOI = 1) for 18 h as indicated and the level of cleaved caspase3 and caspase8 were examined by Western blotting. Results are presented as mean ± SD of four biological replicates (**b**–**e**) (One-way ANOVA followed by Bonferroni multiple comparison). One representative experiment of three is shown. n.s., no significance; ****P < 0.0001.

Since HECTD3 deficiency caused reduced virus replication, impaired innate response, and decreased K33-linked PKR polyubiquitination, we hypothesized that the roles of HECTD3 in innate antiviral response might rely on its effects on polyubiquitination of PKR. By using *Pkr*^*−/−*^ RAW264.7 cells generated by Crisper-Cas9 system and reconstituted with wild-type PKR (wt) or mutant PKR (K68R) (Fig. 7a), we found PKR deficiency significantly promoted the replication of VSV and PKR (wt) or PKR (K68R) could both rescue the anti-replication capacity while knockdown of HECTD3 no longer had the ability to inhibit virus replication in *Pkr*^*−/−*^ RAW264.7 cells and *Pkr*^*−/−*^ cells reconstituted with mutant PKR (K68R) (Supplementary Fig. 9a and Fig. 7b). On the other hand, the IL-6 and IFNβ production in control siRNA or *Hectd3* siRNA transfected *Pkr*^*−/−*^ RAW264.7 cells were comparable after infected by VSV and only PKR (wt) but not mutant PKR (K68R) could restore IL-6 and IFNβ production (Fig. 7c, d). Moreover, PKR deficiency could rescue the VSV-induced cleavage of Caspase8/Caspase3 and cell apoptosis, which were promoted by knocking down *Hectd3* in wild-type RAW264.7 cells (Fig. 7e, f). These results suggested that PKR is critical for HECTD3-mediated anti-viral response against RNA virus, and PKR (K68R) mutant, which cannot be ubiquitinated by HECTD3 tends to inhibit viral replication rather than mediate the production of inflammatory cytokines.

### HECTD3 promotes virus-induced formation of PKR-IKK complex and activation of IKK signaling

**Fig. 8:**
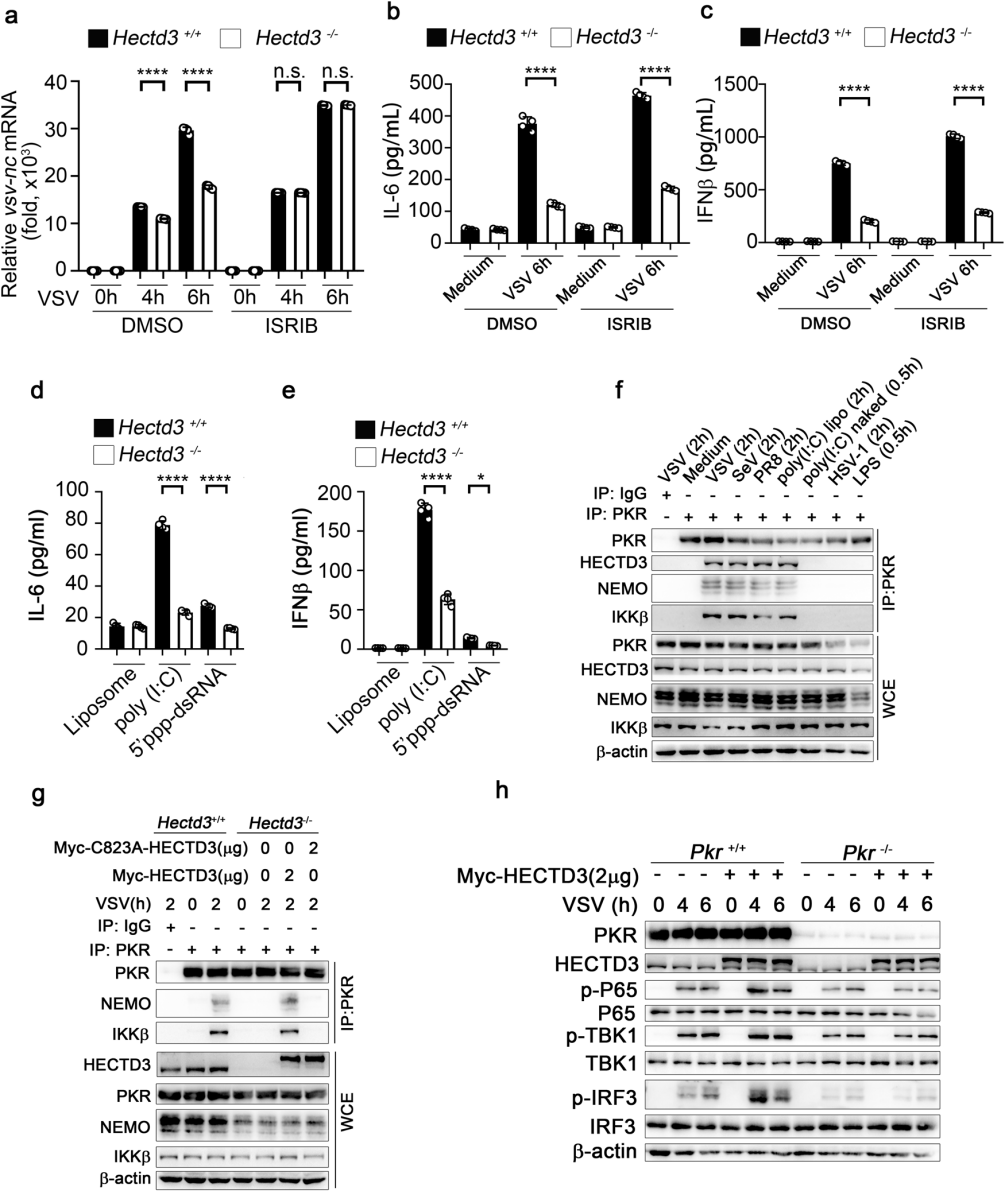
K33-linked PKR forms a complex with IKK and exerts proinflammatory function. **a**–**c** *Hectd3*^*+/+*^ or *Hectd3*^*–/–*^ BMDM (2 × 10^5^ cells per 24-well) were pretreated with DMSO or ISRIB (3.5 nM) for 1 h and infected with VSV (MOI = 1) for 4 h, 6 h as indicated. The mRNA level of VSV nucleocapsid (VSV-nc) gene was examined by Q-PCR (**a**) and the amounts of IL-6 (**b**) and IFNβ (**c**) in supernatants were measured by ELISA. **d**, **e** *Hectd3*^*+/+*^ or *Hectd3*^*–/–*^ BMDM (2 × 10^5^ cells per 24-well) were stimulated with cytosolic nucleic acids sensor agonists for 6 h (500 ng/ml 5’ppp-dsRNA for RIG-I, 500 ng/ml poly (I:C) for MDA5). The amounts of IL-6 (**d**) and IFNβ (**e**) in supernatants were measured by ELISA. **f** Wild-type BMDM were infected with VSV (MOI = 1), SeV (MOI = 1), PR8 (MOI = 1), stimulated with liposome-packaged poly (I:C) (1 µg/ml), naked poly (I:C) (5 µg/ml), HSV-1 (MOI = 5) or LPS (100 ng/ml) for 0.5 h or 2 h. Then whole-cell extracts (WCE) were immunoprecipitated with anti-PKR antibody plus protein A/G beads. Components in the PKR complex were examined by Western blotting. **g** *Hectd3*^+/+^ or *Hectd3*^–/–^ Raw264.7 cells reconstituted by Mock, Myc-HECTD3 or Myc-HECTD3 (C823A) were infected with VSV (MOI = 1) for 2 h as indicated. Then whole-cell extracts (WCE) were immunoprecipitated with anti-PKR antibody plus protein A/G beads. Components in the PKR complex were examined by Western blotting. **h** *Pkr*^*+/+*^ *or Pkr*^*–/–*^ Raw264.7 cells were transfected with a plasmid expressing Myc-HECTD3 and infected with VSV (MOI = 1) for 4 h or 6 h as indicated. The activation of the signaling mediators were examined by Western blotting. Results are presented as mean ± SD of three biological replicates (**a**–**e**) (one-way ANOVA followed by Bonferroni multiple comparison). One representative experiment of three is shown. n.s., no significance; *P < 0.05; ****P < 0.0001.

The multifunction of PRRs and the complex interplay between PRRs and/or other immune pathways finely tunes the outcome of host immune defence responses [20, 21]. Previous studies have demonstrated that in addition to regulating the translation of viral proteins as mentioned above, PKR also regulates the activation of anti-viral innate signaling and the production of relevant cytokines [7]. We also found that PKR deficiency promoted viral propagation while inhibited the inflammatory responses which cannot influenced by *Hectd3* siRNA. These data suggested that HECTD3 may enhance the proinflammatory function of PKR in innate anti-viral response. We first used ISRIB, which was a commonly used inhibitor of EIF2α [22, 23], to elucidate whether PKR-EIF2α could participate in the HECTD3-promoted inflammatory innate response. In our study, 3.5 nM ISRIB significantly inhibited the phosphorylation of EIF2α but had no obvious effect on PKR phosphorylation (Supplementary Fig. 9b). We found that ISRIB abrogated the anti-viral replication capacity (Fig. 8a) but not the inhibition of IL-6 and IFNβ production by HECTD3-deficient in BMDM infected by VSV (Fig. 8b, c). Our further study found the reduced production of IL-6 and IFNβ in HECTD3-deficient BMDMs in response to cytosolic RNA sensor agonists (5’ppp-dsRNA and poly (I:C)) which lack replication capacity (Fig. 8d, e). These results suggested that the proinflammatory effect of HECTD3 may also dependent on PKR’s proinflammatory function except for the inhibition of PKR-EIF2α-mediated restriction of viral replication. PKR had been reported to interact with the IKK complex to activate NF-κB signal [24]. In our work, we found PKR can form complexes with IKKβ and NEMO in BMDMs upon RNA virus infection but not DNA virus infection or TLR agonist treatment (Fig. 8f). The interactions between PKR and IKK-related kinases were impaired in *Hectd3*^*−/−*^ RAW264.7 macrophages infected with RNA viruses, while reconstitution of HECTD3 but not the HECTD3 (C823A) mutant rescued the formation of PKR-IKK complex (Fig. 8g). This suggests that the binding of the PKR and IKK complex is dependent on the E3 ubiquitin ligase activity of HECTD3.

By using *Pkr*^+/+^ and *Pkr*^*−/−*^ RAW264.7 cells and overexpressing HECTD3, we found that PKR deficiency inhibited the anti-RNA virus innate response as well as abrogated HECTD3-mediated enhancement of NF-κB and TBK1-IRF3 signal activation (Fig. 8h) and subsequent IL-6 and IFNβ productions (Supplementary Fig. 10a, b). The production of IL-6 and IFNβ were also impaired in *Pkr*^*−/−*^ RAW264.7 cells transfected with RNA mimic poly(I:C), which could not be rescued by HECTD3 overexpression (Supplementary Fig. 10c, d). These results suggested that HECTD3 enhanced the innate inflammatory response through promoting the formation of PKR-IKK complex via catalyzed K33-linked PKR.

## Discussion

Hosts eliminate RNA virus infections by inhibiting viral replication and producing inflammatory cytokines, interferons, and these two host defense responses must be finely tuned in order to avoid both viral spreading and lethal inflammation [25, 26]. Our findings demonstrate that HECTD3 deficiency can accelerate virus clearance while restrain virus-induced inflammation. Moreover, HECTD3 inhibits PKR’s anti-replication ability but promotes PKR’s proinflammatory function against RNA virus through ubiquitination. These results propose HECTD3 as a potential target for controlling RNA virus infection and virus-induced inflammatory damage once pharmacologically inhibited or degraded.

Protein ubiquitination catalyzed by E3 ligases plays essential roles in the regulation of innate anti-viral and inflammatory responses [27]. However, the role and the mechanism of HECTD3 in infection and inflammation have not been fully elucidated. HECTD3 was initially found to regulate cell proliferation and cell death by regulating the ubiquitination of Tara, caspase8 and caspase9 in the context of tumor research [28]. Recently, HECTD3 is implicated as an E3 ubiquitin ligase for TRAF3 and STAT1 in bacterial infection [16], hypothermic oxygenated perfusion of liver and cardiac inflammation models [29]. No report has been published to date about the role of HECTD3 in the antiviral innate immune response. Our study demonstrated a critical role in controlling innate immune response to RNA viruses for HECTD3, thus clarifying the immune function of HECTD3 during RNA virus infection. Various E3 ligases had been reported to regulate virus-induced inflammatory response and viral replication separately via targeting various substrates. Compared to other E3 ligases, HECTD3’s unique function is that it simultaneously promotes the replication of RNA viruses and the production of inflammatory cytokines. HECTD3 is so far the only E3 ligase mediating the polyubiquitination of PKR that is not associated with the degradation of PKR. These results indicate that the downregulation of HECTD3 during RNA virus infection is a protective mechanism by host to not only eliminate the infection but also avoid excessive inflammation. Nevertheless, the mechanism by which host downregulates the expression of HECTD3 in response to RNA virus infection still awaits further investigation.

HECTD3 has been reported to mediate the innate immune response to bacterial infection via promoting the ubiquitination of TRAF3 [16], therefore one obvious question is whether TRAF3 is also the substrate of HECTD3 that mediates the anti-RNA virus response. Our study shows that HECTD3 binds to PKR in macrophages, which is enhanced after RNA virus infection, and that TRAF3 is absent from this HECTD3-PKR complex during this process. These results suggest that TRAF3 is not involved in HECTD3-mediated effect in RNA virus infection model. Our study thus provides mechanistic insights for the role of HECTD3 in enhancing viral replication and inflammatory signal activation by linking K33 polyubiquitin chain to PKR. However, which upstream signal regulates the PKR polyubiquitination mediated by HECTD3 requires further elucidation.

Accumulating studies have revealed important roles of PKR in anti-viral innate responses [30]. The classic function of PKR is to activate EIF2α and inhibit the translation of viral proteins upon dimerization and autophosphorylation after recognizing dsRNA, a typical by-product of RNA virus replication. Prolonged PKR activation also can promote cell apoptosis in the late stage of infection. These two functions are effector mechanisms that critically limit viral spread in an infected host [31]. Our work provides the evidence that K33-linked polyubiquitination of PKR by HECTD3 inhibits the dimerization and subsequent activation of PKR. Previous studies found that the activation of latent PKR requires its dimerization and autophosphorylation that occur upon PKR recognizing sufficiently long dsRNA, which is inhibited by shorter dsRNA (15–30 bp) through competitive binding [32, 33]. PKR can also autophosphorylate if its concentration is high enough [34]. These suggest that the structural accessibility is the key to the dimerization and autophosphorylation of PKR. Our work shows that HECTD3 binds to the PKR N-terminal region and catalyzed Lys68 site for ubiquitination which is located in dsRBM motif of PKR. Hence, we hypothesize that the K33-linked polyubiquitination catalyzed by HECTD3 forms a structure in dsRBM motif that hinders the dimerization and subsequent activation of PKR. However, the specific mechanism still needs further investigation.

Previous studies have found that PKR can promote the production of inflammatory factors and type I interferons by promoting the activation of IRF3 and MAPK signaling [8–11, 35]. PKR has also been reported to regulate the inflammatory response by promoting NF-κB activation through the activation of NIK and IKKβ and the IFN response by stabilizing the IFN-β mRNA [36, 37]. But how these proinflammatory functions of PKR are regulated during RNA virus infection remain unclear. In our study, we found that the K33-linked PKR catalyzed by HECTD3 inhibited the dimerization and activation of PKR but enhanced the binding of PKR to IKKβ and NEMO and promoted the activation of downstream signals in response to RNA virus. These results indicate that K33-linked PKR, though limits its own dimerization and phosphorylation, provides a structural basis for its binding to the IKK complex and then promote the activation of IKK signalosome irrespective of its kinase activity. Consistent with our conclusions, some other groups found that PKR activates NF-κB independent of its kinase activity and its binding to EIF2α [24, 38, 39]. We also find that VSV infection downregulates the expression of HECTD3 in macrophages under physiological conditions. This suggests that HECTD3 can promote the proinflammatory effect of PKR at the early stage of virus infection and thus alert surround cells. At the late stage of infection, the downregulation of HECTD3 enables the dimerization of PKR and thus suppresses virus replication while avoiding excessive inflammation. Our work shows that HECTD3 is required for the finely tuned immune response against RNA viruses and HECTD3 is a potential target for the treatment of RNA virus infection and associated inflammation.

## Materials and methods

### Mice and cells

6–8 weeks of age C57BL/6 (H-2K^b^) WT mice were from Joint Ventures Sipper BK Experimental Animal (Shanghai, China). The *Hectd3* conditional knockout mice were established by using a recombinant strategy through replacing exon 3 and exon 4 of *Hectd3* with insertion of LoxP sequence at both ends, and were backcrossed with C57BL/6 mice for more than six generations for experiments. The *Hectd3*^flox/flox^ mice were crossed with EIIA-Cre mice to create complete knockout mice (*Hectd3*^**−/−**^) for experiments. For genotyping of mice, tail DNA was extracted, and PCR was performed as described. The genotyping primer sequences and the standards were described in (Supplementary Table 1). All animal experiments were undertaken in accordance with the National Institute of Health Guide for the Care and Use of Laboratory Animals, with the approval of the Scientific Investigation Board of Second Military Medical University, Shanghai.

HEK293T, L929, THP1, and RAW264.7 cells were obtained from and authenticated by the American Type Culture Collection (Manassas, VA), and were cultured as suggested by supplier. PMs and BMDMs were isolated or prepared and cultured as described previously [19]. For the depletion of mouse HECTD3 or PKR in RAW264.7 and L929 cells, pc3-U6-guide RNA-CMV-RED (encoding guiding RNA and red fluorescent protein–RFP) and Cas9-IRES-EGFP (encoding Cas9 and green fluorescent protein–GFP) plasmids (kind gifts from Shanghai Model Organisms Center & Inc, Shanghai, China) were cotransfected into RAW264.7 and L929 cells as described. Cells with both red and green fluorescence were then sorted by using Gallios Flow Cytometer (Beckman Coulter, Brea, CA). Sorted cells were cultured for 3–5 days, and clones propagated from single cell were picked out. Three target sequences for guiding RNA synthesis against *Hectd3* and *Pkr* were designed (Supplementary Table 1). Fluorescent quantitative PCR (qRT-PCR) and primers for mycoplasma (Forward: GGGAGCAAACAGGATTAGATACCCT and Reverse: TGCACCATCTGTCACTCTGTTAACCTC) were used to determine whether cells had mycoplasma contamination.

### Antibodies, reagents, and viruses

The antibodies used in this study were listed in (Supplementary Table 2). VSV (Indian strain, a gift from Dr. Weiqing Pan, Second Military Medical University), HSV-1 (F strain, a gift from Dr. Qihan Li, Chinese Academy of Medical Sciences, China) and VACV (ATCC, Manassas, VA) were propagated in the monolayer of Vero cells. SeV (a gift from Dr. Bin Sun, Shanghai Institutes for Biological Sciences, Chinese Academy of Sciences, China) and PR8 (was generated by reverse genetics as previously described) [40] were propagated by allantoic inoculation of embryonated hen’s eggs. For infection in vitro, cells were infected with VSV (MOI = 1), SeV (MOI = 1), HSV-1 (MOI = 5) or VACV (MOI = 5) respectively. Poly (dA:dT) (5 μg/ml), ISD (1 μg/ml), 5’ppp-dsRNA (1 μg/ml) and poly(I:C) (1 μg/ml) were from InvivoGen (San Diego, California). Act D, MG132, ISRIB were purchased from Selleck. Other non-specified reagents were purchased from Sigma-Aldrich (St. Louis, MO). Cells infected with VSV(MOI = 1) for 1 h, the amount of virus in the cells represent the amount of virus entry. While incubated at ice-cold temperature, virus can only attach to the cells but not enter the cells and the amount of virus adhered to cells represent the amount of virus attachment [41]. Amounts of VSV and HSV-1 were detected using VSV nucleocapsid(nc) and HSV-1 ICP0 q-PCR assay.

### Plasmids, transfection and RNA interference

The recombinant vectors encoding mouse HECTD3 (GenBank No. NM_175244.4), PKR (GenBank No. NM_011163.4) and the indicated mutations were constructed as described. For transient transfection of plasmids in RAW264.7 and HEK293T cells, the X-tremeGENE HP reagents were used according to manufacturer’s instructions (Roche, Welwyn Garden City, UK). For transient knockdown of Hectd3, three siRNA duplexes were synthesized (Supplementary Table 1) and transfected using the INTERFERin-HTS according to the standard protocol (Polyplus-transfection Company, Illkirch, France). The non-sense sequence 5′-TTCTCCGAACGTGTCACG-3′ was used as control siRNA.

### Quantitative-PCR

Total cellular RNA was extracted using Trizol reagent (Invitrogen Corporation, CA, USA), and cDNA was synthesized by using the AMV Reverse Transcriptase kit (Promega, Madison, WI). The mRNA quantification methods were as described [42, 43]. The primer sequences were listed in (Supplementary Table 1).

### ELISA

ELISA kits for mouse IFNβ, IL-6 were from R&D Systems (Minneapolis, MN). The concentrations of cytokines in the culture supernatants or serum were determined as recommended by the manufacturer.

### Nano spray liquid chromatography–tandem mass spectrometry

HEK293T cells were transfected with Myc-HECTD3 for 48 h and infected with or without VSV. Then whole cells lysates were immunoprecipitated (IP) with anti-Myc Sepharose Beads. The immune complexes were separated by one-dimensional SDS-PAGE and the gel was stained with Coomassie blue. Bands presented differently between lysates from infected and uninfected cells were subjected to MS assays. The nano-ultra performance liquid chromatography–electrospray ionization tandem MS was performed by the Beijing Protein Innovation Co., Ltd (Beijing, China).

### Immunoprecipitation and immunoblot

The immunoprecipitations using anti-HECTD3, anti-PKR, anti-Flag or anti-Myc antibodies and the immunoblot assays were performed as described previously [19, 42, 43]. Full length uncropped original western blots are available in Supplemental Material.

### Polyubiquitination assays

For the examination of polyubiquitination of PKR, cells were lysed in stringent lysis buffer supplemented with 1% Triton X-100, 1% NP-40 and 300 mM NaCl. Lysates were denatured by heat in 1% SDS, followed by immunoprecipitations with anti-PKR antibody plus protein A/G beads. The pellets were extensively washed for four times with the lysis buffer. Then the final pellets were resolved in SDS sample buffer, and the ubiquitination of PKR was examined by Western blot.

### Immunofluorescence confocal microscopy

RAW264.7 cells seeded on cover sliders were transiently co-transfected with HECTD3-Nred for 36 h. Samples were washed briefly in PBS and fixed in 4% paraformaldehyde. For colocalization assays of HECTD3 and PKR, cells were permeabilized in blocking buffer containing 0.5% saponin, and then sequentially stained with anti-PKR primary antibody and Oregon Green 488–conjugated secondary antibody. Upon confocal microscopy, cells were immediately stained with 10 μg/ml DAPI and covered by cover glasses. Images were obtained with a laser scanning confocal microscope (Leica TCS SP8) and analyzed by the LAS X software version 2.0.2.15022.

### Flow cytometry

The BMDM or RAW267.4 cell infected by VSV were harvested and wash by PBS. Cells were stained with ANNEXIN V-FITC at room temperature for 15 min and the percentage of FITC positive cells was analyzed by flow cytometry as described using a LSR II flow cytometer (BD Biosciences, New Jersey, NJ). For flow cytometry staining, single-cell suspensions from spleen and lymph nodes were stained with the indicated antibodies at room temperature (RT) for 20 min, including CD4^+^ T cells (CD45^+^ CD4^+^), CD8^+^ T cells (CD45^+^ CD8^+^), B cells (CD3^−^ CD19^+^), macrophages (CD45^+^ CD11b^+^ F4/80^+^), neutrophils (Ly6G^+^ CD11b^+^), natural killer (NK) cells (CD45^+^ NK1.1^+^), and dendritic cells (CD11c^+^ I-A[b]^+^). The cells were washed twice and analyzed by flow cytometry on BD LSR Fortessa (BD Biosciences) with FlowJo software.

### Animal models and manipulations

Age- and gender-matched mice (8 weeks) with indicated genotype were randomly sellected to establish animal models. For in vivo infection with pathogens, mice were intraperitoneally injected with 100 μl PBS containing 2 × 10^7^ PFUs of VSV. The liver and lung were excised, fixed in 4% formalin, embedded in paraffin, cut into 4 μm sections, affixed onto glass slides, de-paraffinized and stained with Hematoxylin and Eosin. Inflammatory cell foci were characterized by immunofluorescence microscopy as described previously and the fluorescent antibodies were listed in (Supplementary Table 2). The titers of VSV in the liver and lung were evaluated by TCID50 assay in BHK-21 cell.

### Statistical analysis

All the experiments were independently repeated at least three times and *n* ≥ 3 in animal studies. Results are given as mean ± SE or mean ± SD. Comparisons between two groups were done using Mann–Whitney *U* test or unpaired Student’s *t* test. The survival curve was established by the Kaplan and Meier method, and analyzed by Log-rank test. Multiple comparisons were done with one-way ANOVA followed by Bonferroni multiple comparisons. Statistical significance was determined as *P* < 0.05.

## Supplementary information

Full and uncropped western blots

Supplementary materials

Checklist

## Data Availability

The authors declare that there are no primary datasets and computer codes associated with this study. All data and materials are available to the researchers once published.
