# Supplementary material for: E3 ligase HECTD3 promotes RNA virus replication and virus-induced inflammation via K33-linked polyubiquitination of PKR
Source: Cell Death Dis. 2023 Jul 4;14(7):396. doi: 10.1038/s41419-023-05923-9 (PMC10319860; doi:10.1038/s41419-023-05923-9)
Supplement: Supplementary file 1 — Full and uncropped western blots [file 41419_2023_5923_MOESM1_ESM.pdf]

Fig. 3a

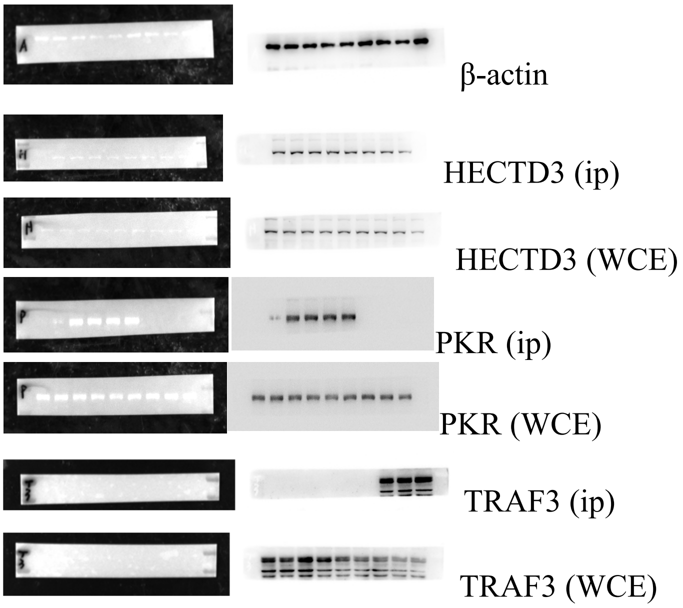
β-actin
HECTD3 (ip)
HECTD3 (WCE)
PKR (ip)
PKR (WCE)
TRAF3 (ip)
TRAF3 (WCE)

Fig. 3b

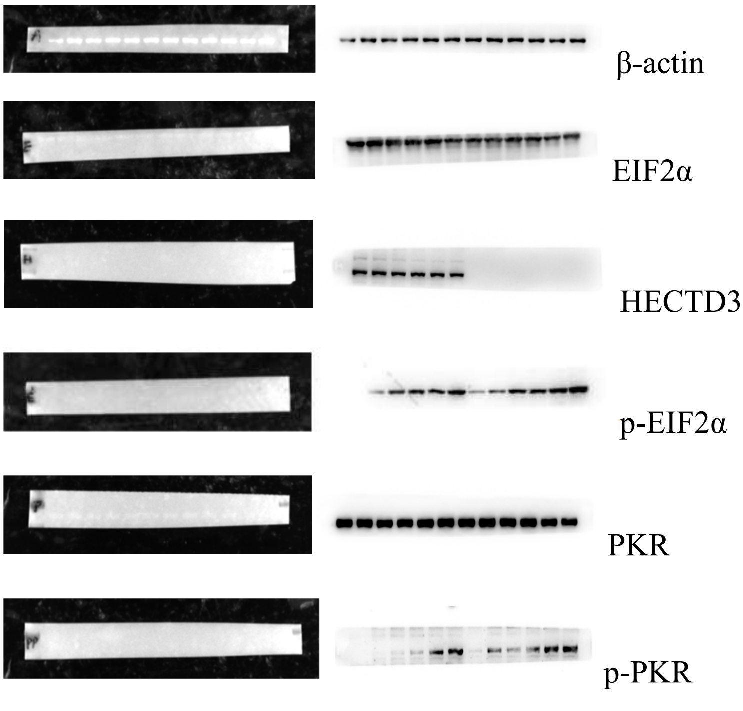
β-actin
EIF2α
HECTD3
p-EIF2α
PKR
p-PKR

Fig. 3c

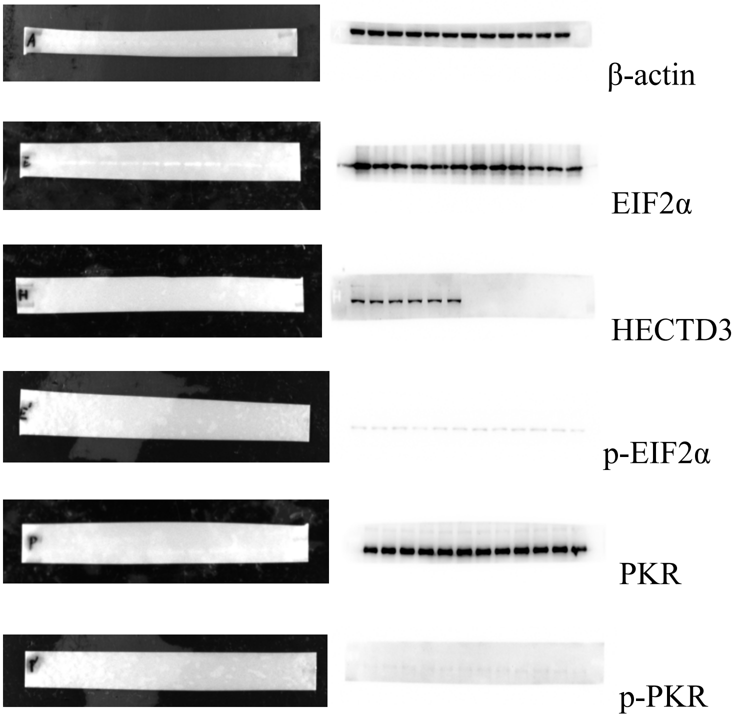
β-actin
EIF2α
HECTD3
p-EIF2α
PKR
p-PKR

Fig. 3d

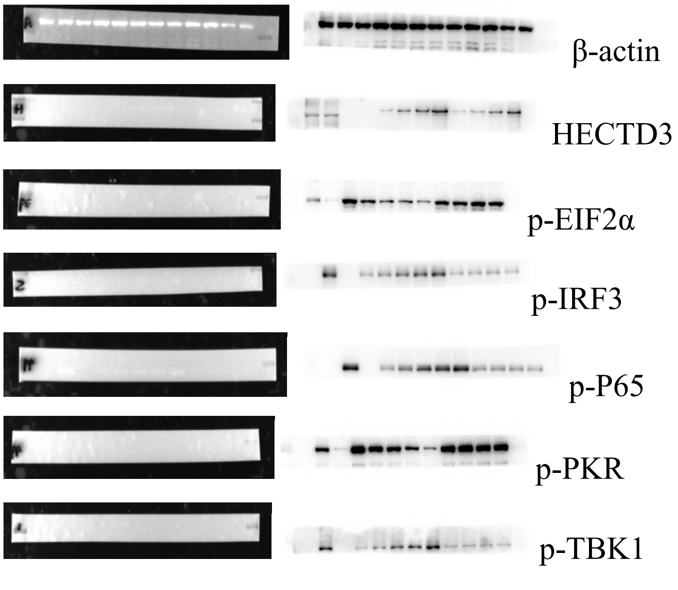
β-actin
HECTD3
p-EIF2α
p-IRF3
p-P65
p-PKR
p-TBK1

Fig. 4b

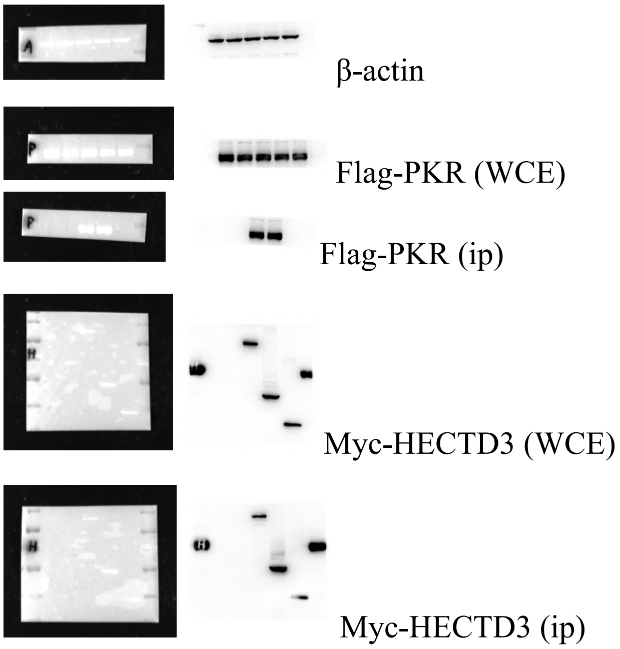
β-actin
Flag-PKR (WCE)
Flag-PKR (ip)
Myc-HECTD3 (WCE)
Myc-HECTD3 (ip)

Fig. 4c

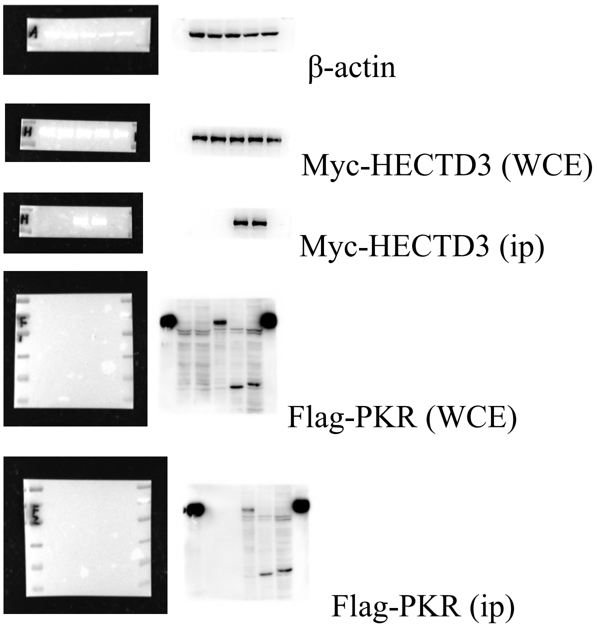
β-actin
Myc-HECTD3 (WCE)
Myc-HECTD3 (ip)
Flag-PKR (WCE)
Flag-PKR (ip)

Fig. 5a

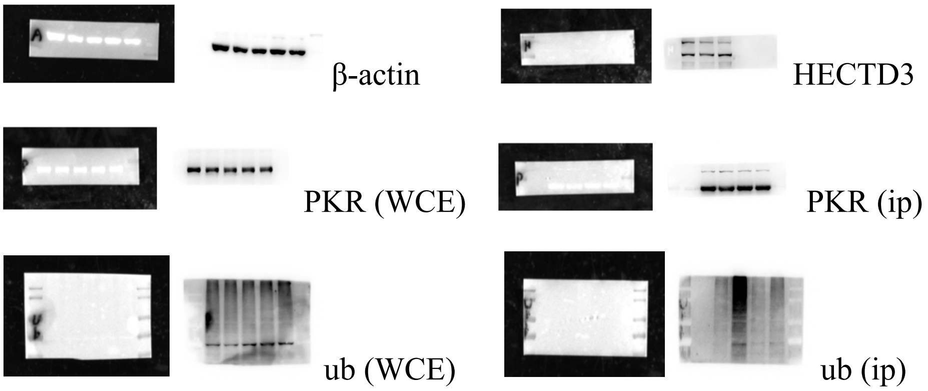

Fig. 5b

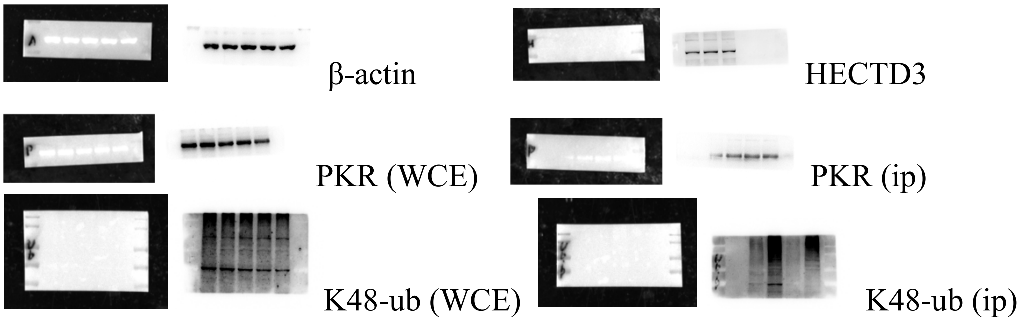

Fig. 5c

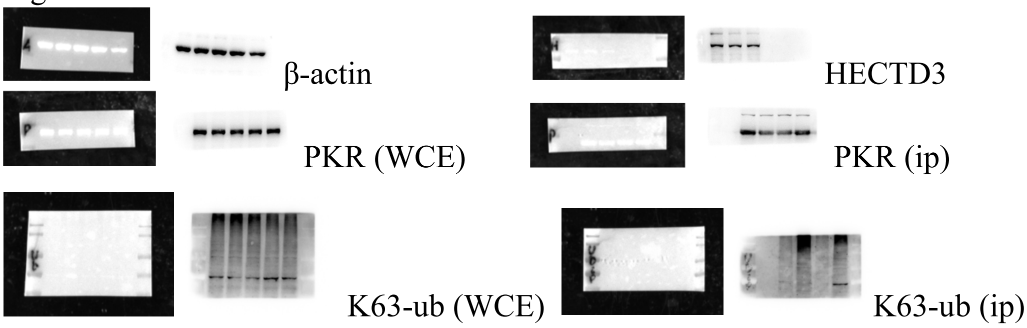

Fig. 5d

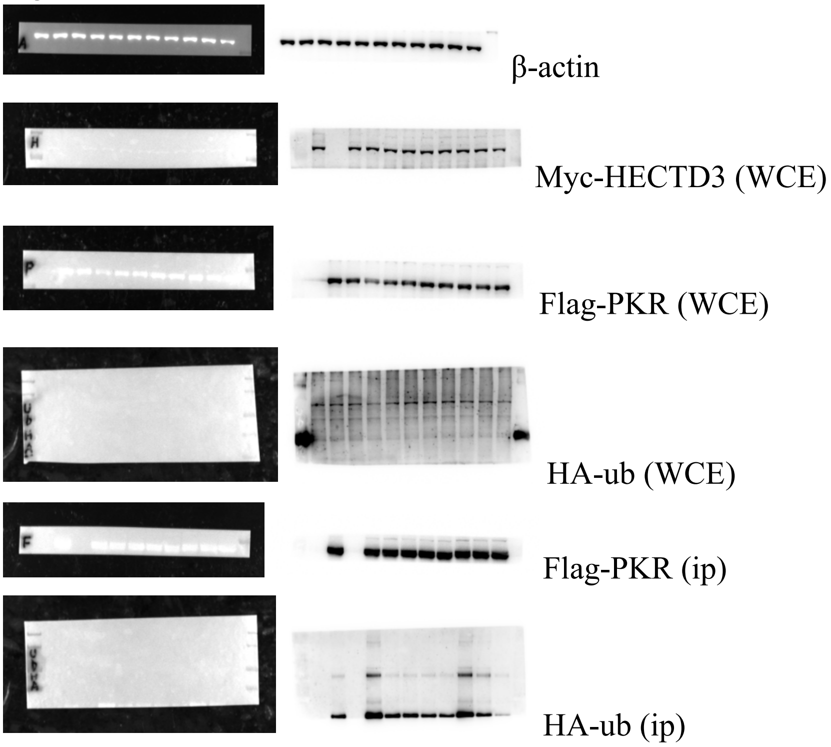
β-actin
Myc-HECTD3 (WCE)
Flag-PKR (WCE)
HA-ub (WCE)
Flag-PKR (ip)
HA-ub (ip)

Fig. 5e

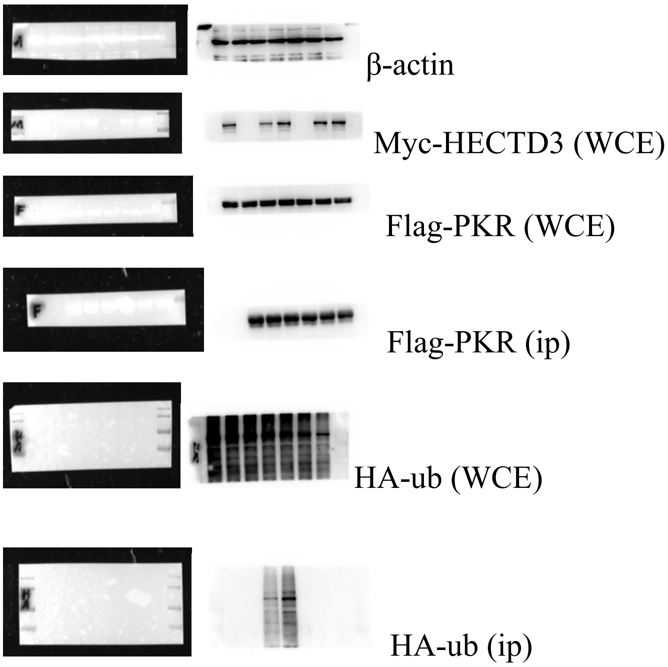
β-actin
Myc-HECTD3 (WCE)
Flag-PKR (WCE)
Flag-PKR (ip)
HA-ub (WCE)
HA-ub (ip)

Fig. 5f

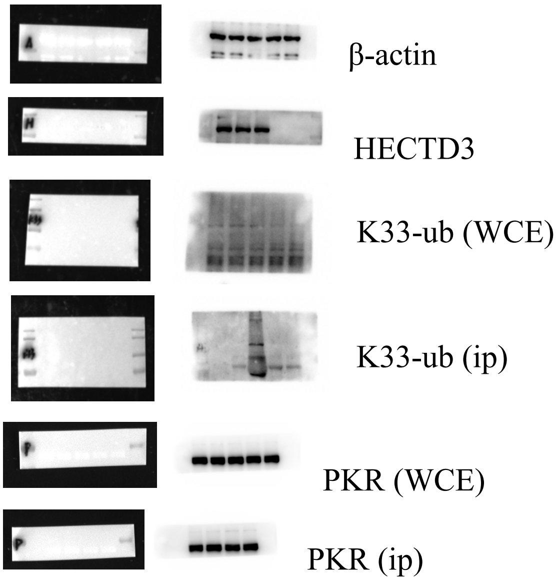

Fig. 6a

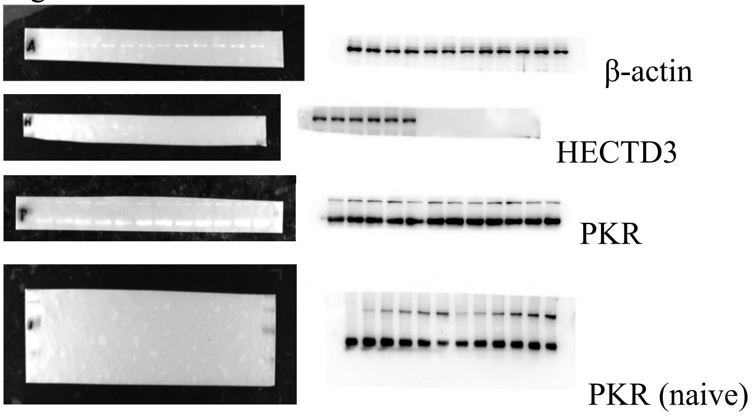

Fig. 6c

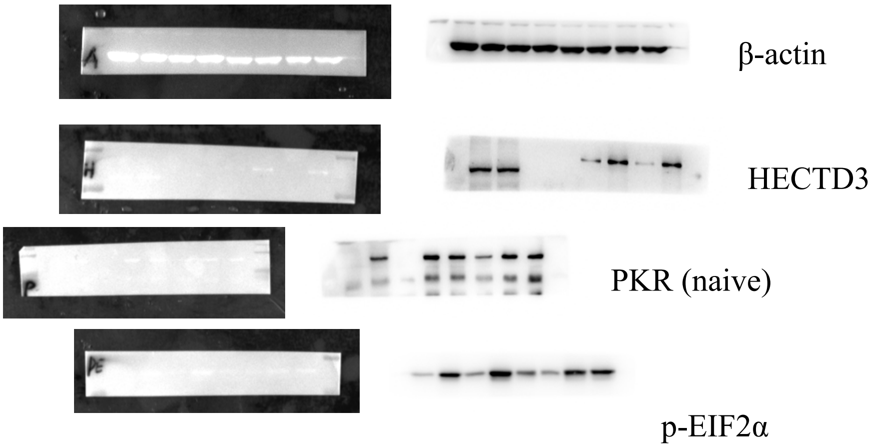

Fig. 7a

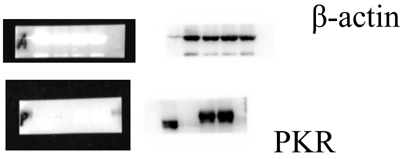

Fig. 7f

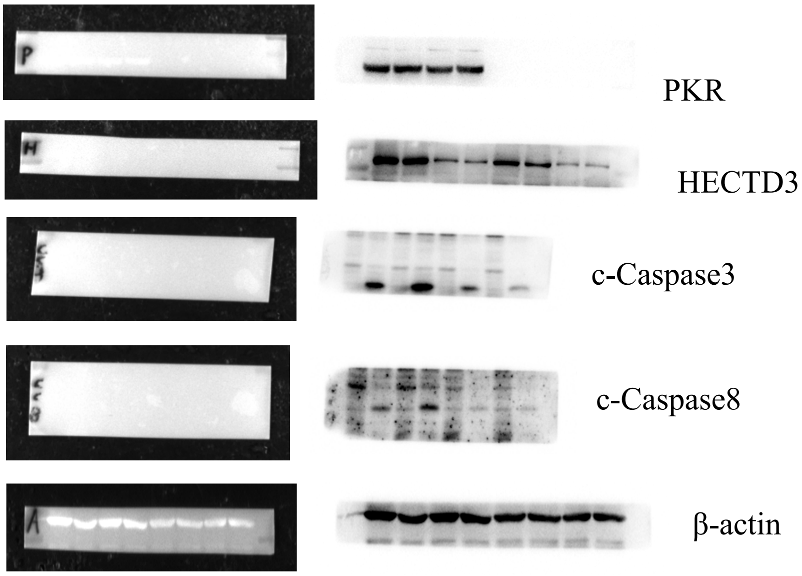

Fig. 8f

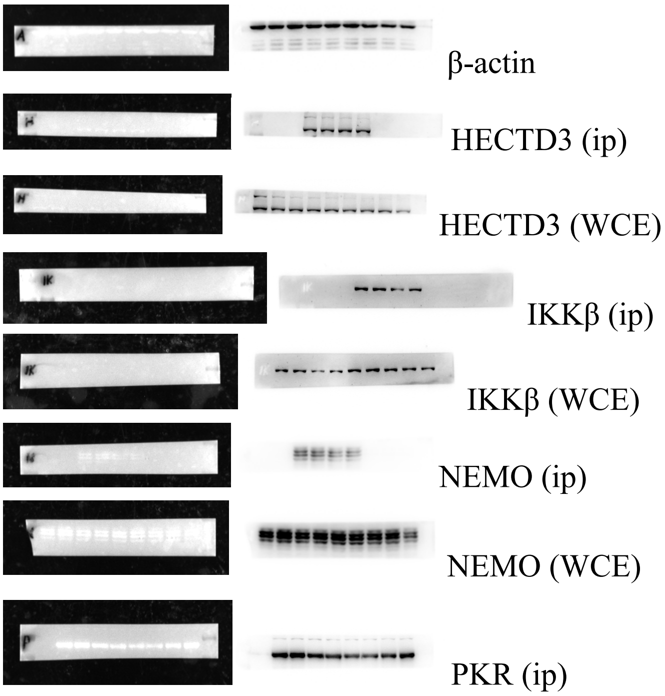

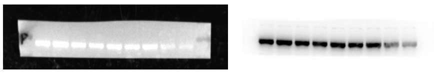

PKR (WCE)

Fig. 8g

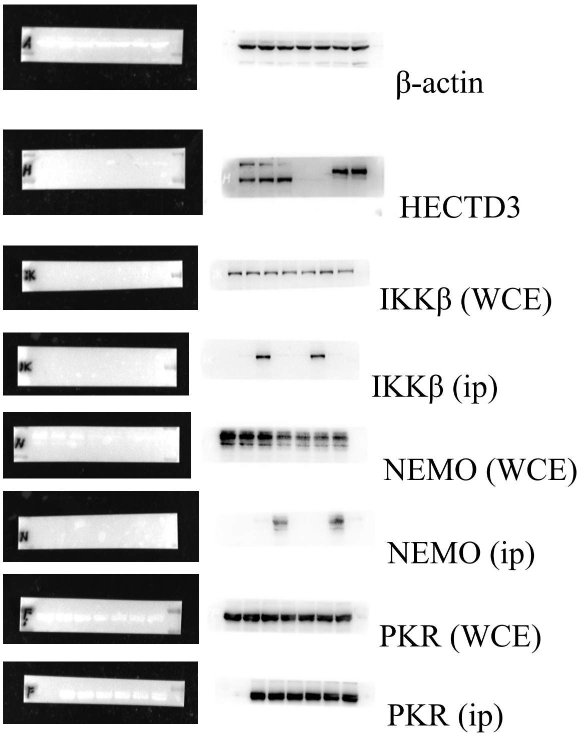
β-actin
HECTD3
IKKβ (WCE)
IKKβ (ip)
NEMO (WCE)
NEMO (ip)
PKR (WCE)
PKR (ip)

Fig. 8h

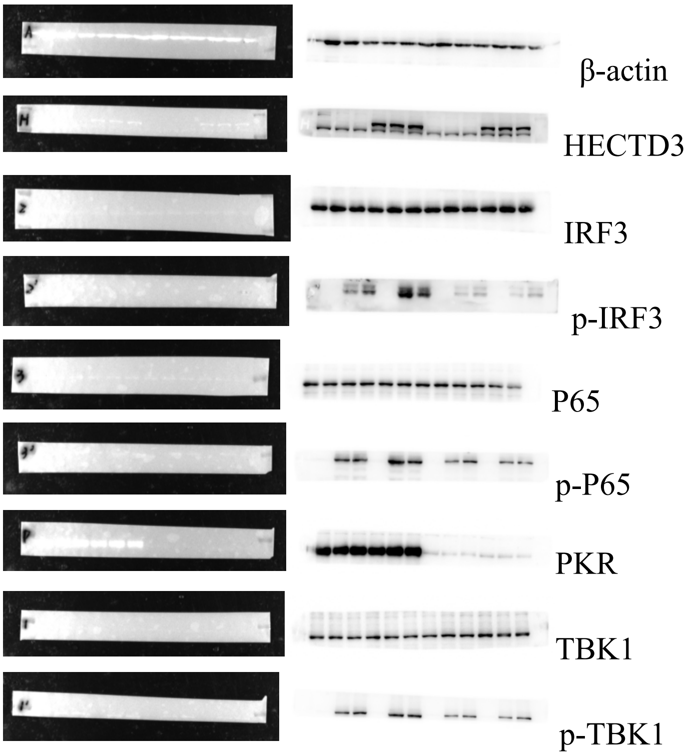
β-actin
HECTD3
IRF3
p-IRF3
P65
p-P65
PKR
TBK1
p-TBK1

Fig. S1b

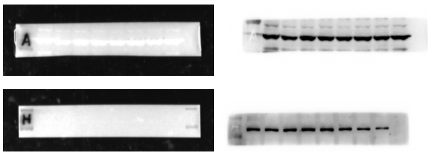

β-actin

HECTD3

Fig. S1c

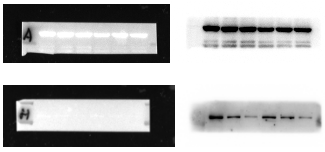

β-actin

HECTD3

Fig. S1d

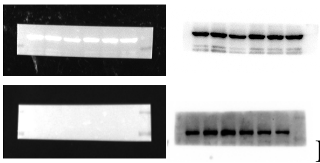

β-actin

HECTD3

Fig. S1e

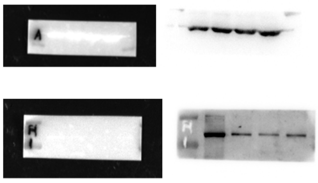

β-actin

HECTD3

Fig. S4a

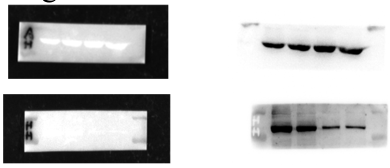

β-actin

HECTD3

Fig. S6a

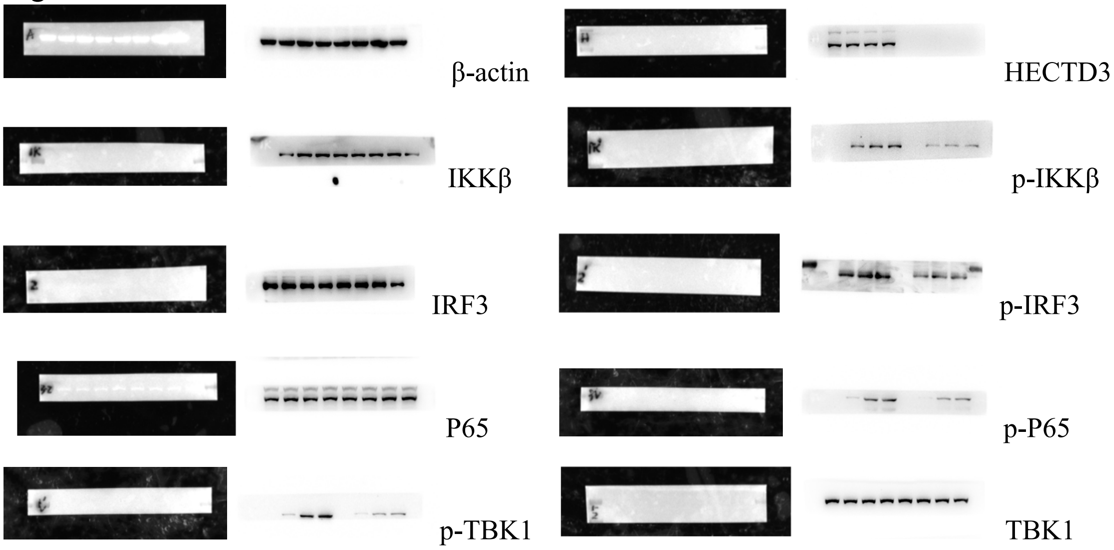

Fig. S6b

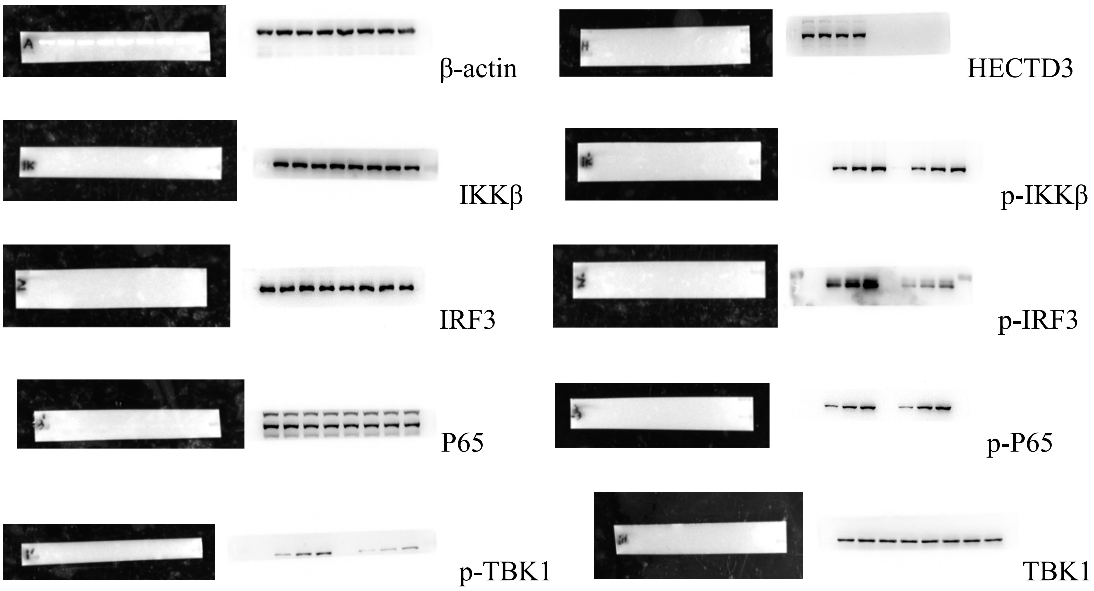

Fig. S7b

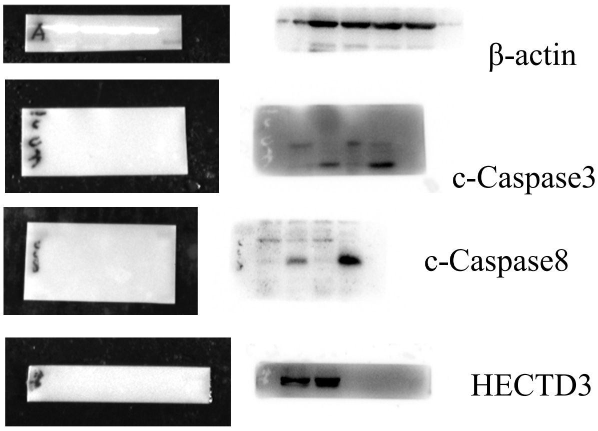

Fig. S8a

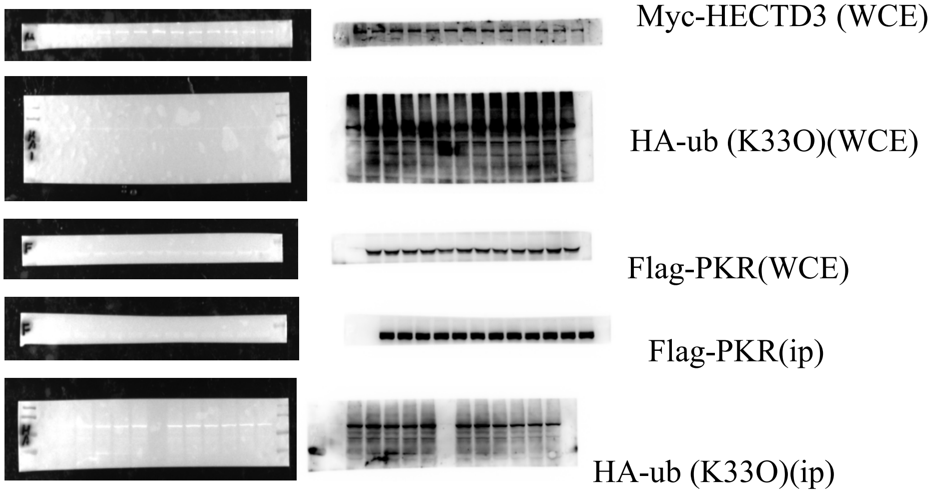

Fig. S8b

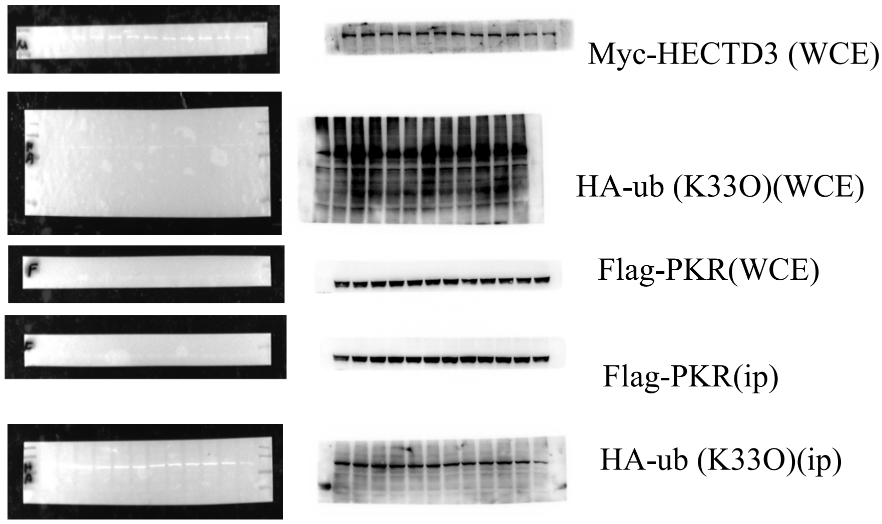

Fig. S8c

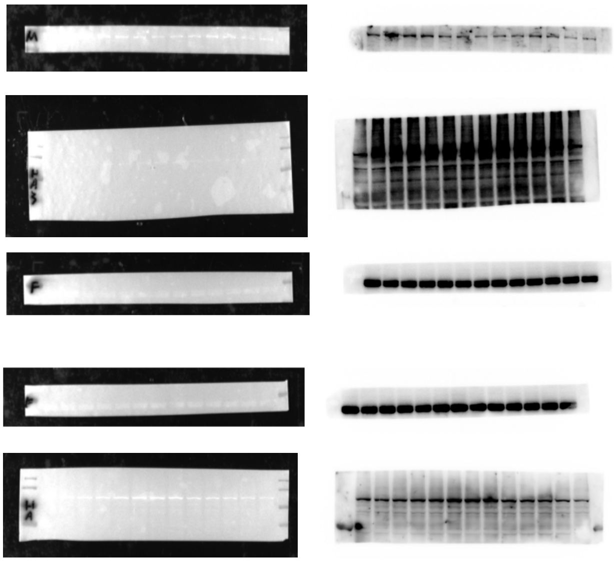

Fig. S9a

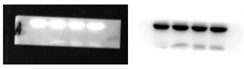

β-actin

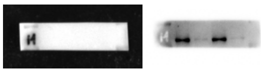

HECTD3

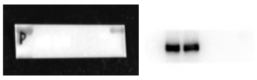

PKR

Fig. S9b

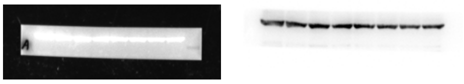

β-actin

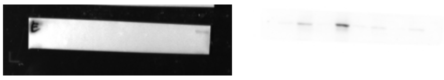

p-EIF2 α

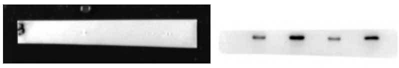

p-PKR

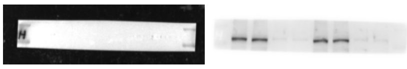

HECTD3
