# Supplementary material for: E3 ligase HECTD3 promotes RNA virus replication and virus-induced inflammation via K33-linked polyubiquitination of PKR
Source: Cell Death Dis. 2023 Jul 4;14(7):396. doi: 10.1038/s41419-023-05923-9 (PMC10319860; doi:10.1038/s41419-023-05923-9)
Supplement: Supplementary file 2 — Supplementary materials [file 41419_2023_5923_MOESM2_ESM.docx]

**Supplementary material**

**E3 ligase HECTD3 promotes RNA virus replication and virus-induced inflammation via K33-linked polyubiquitination of PKR**

Jiaying Huang, Zhou Yu, Xuelian Li, Mingjin Yang, Qian Fang, Zheng Li, Chunmei Wang, Taoyong Chen & Xuetao Cao

Contents:

10 Supplementary Figures and Legends

2 Supplementary Tables


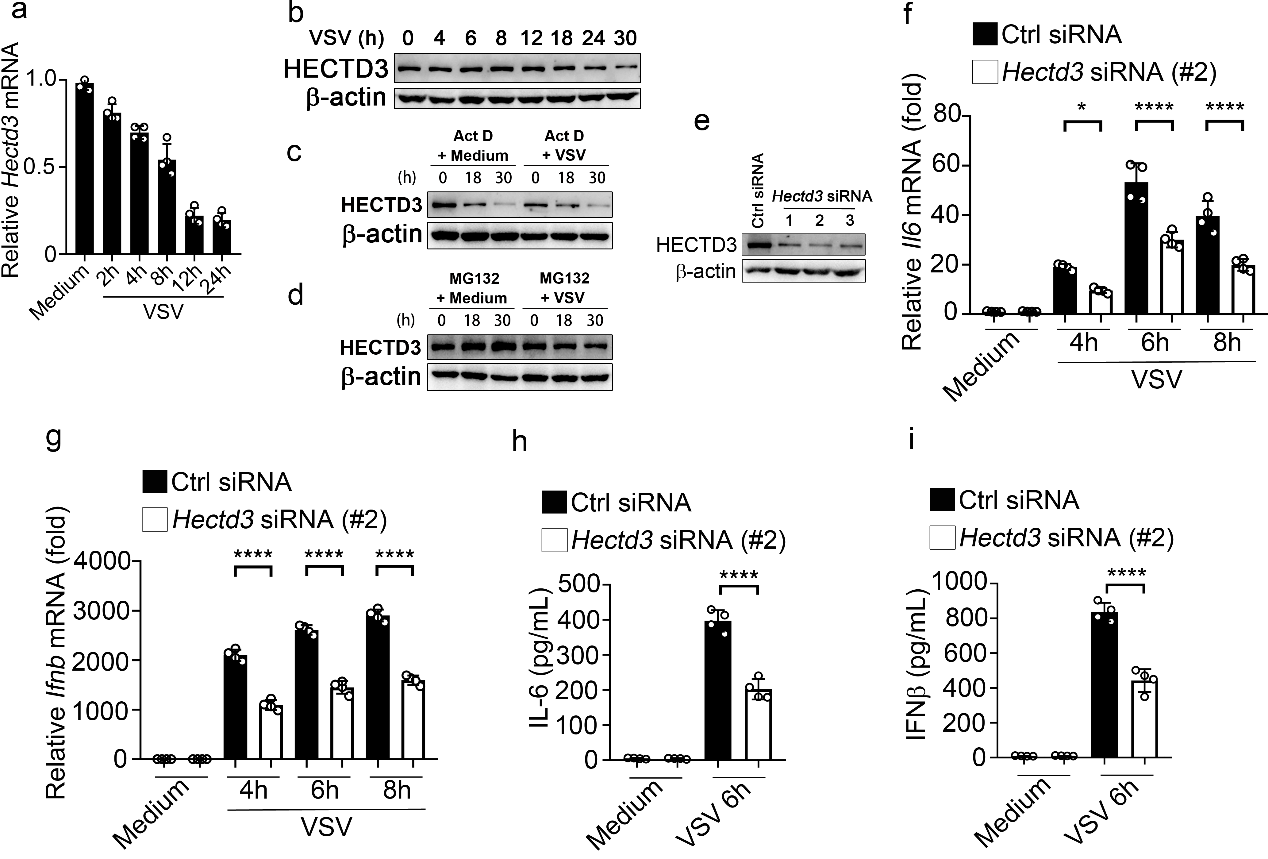


**Supplementary Fig.1** **Knockdown of *Hectd3* impairs innate immune response against VSV in peritoneal macrophages. a, b** Peritoneal macrophage from C57BL/6 mice were infected with VSV (MOI = 1) for indicated periods of time, the mRNA level of *Hectd3* was evaluated by Q-PCR (a), the protein level of HECTD3 was evaluated by Western blotting (b). **c, d** Peritoneal macrophage from C57BL/6 mice were infected with VSV (MOI = 1) as indicated in the presence of actinomycin D (Act D, 10 μM) or MG132 (10 μM), the protein level of HECTD3 was evaluated by Western blotting. **e-i** Peritoneal macrophages from C57BL/6 mice were transfected with control (Ctrl) or *Hectd3*-specific siRNAs for 48h. The efficiency of knockdown (KD) was evaluated by Western blotting (e). The cells were then infected with VSV (MOI = 1) as indicated (f and g) or for 6h (h and i) and the mRNA levels of *Il6* and *Ifnb* were evaluated by Q-PCR (f and g) and amounts of IL-6 and IFNβ in the supernatants were measured by ELISA (h and i). Results are presented as mean ± SD of triplicate samples (a and e-h; one-way ANOVA followed by Bonferroni multiple comparison). One representative data of three independent experiments are shown. *, *P* < 0.05; ****, *P* < 0.0001.

**
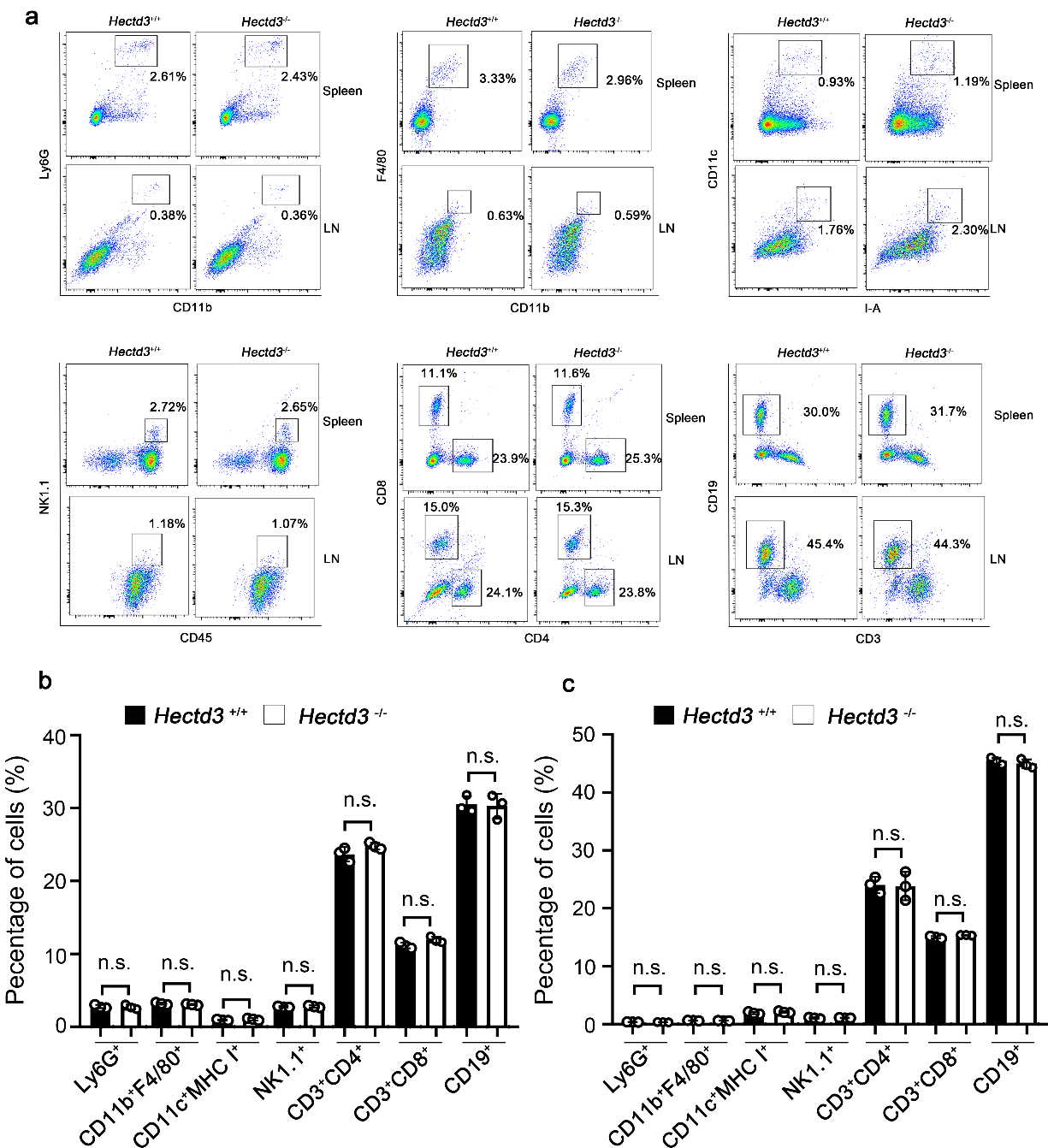
**

**Supplementary Fig.2 HECTD3 deficiency does not affect the distribution of major immune cell populations in secondary lymph organs. a** Single-cell suspensions of spleen and mesenteric lymph nodes of *Hectd3^+/+^* (n = 3) or *Hectd3^–/–^* (n = 3) mice were stained with fluorescent markers and analyzed by FACS. Cells were gated for CD45^+^CD3^+^ cells, and then analyzed for CD4^+^ T cells and CD8^+^ T cells. Otherwise, cells were gated for CD45^+^ cells, and then analyzed for CD19^+^ B cells, Ly6G^+^ Neutrophils, NK1.1^+^ NK cells, CD11b^+^F4/80^+^ macrophages or CD11c^+^I-A[b]^+^ DCs. **b, c** The corresponding cell populations in spleen (b) and lymph nodes (c) were summarized and presented as mean ± SD (One-way ANOVA followed by Bonferroni multiple comparison). n.s., not significant (as compared to *Hectd3^+/+^* group).


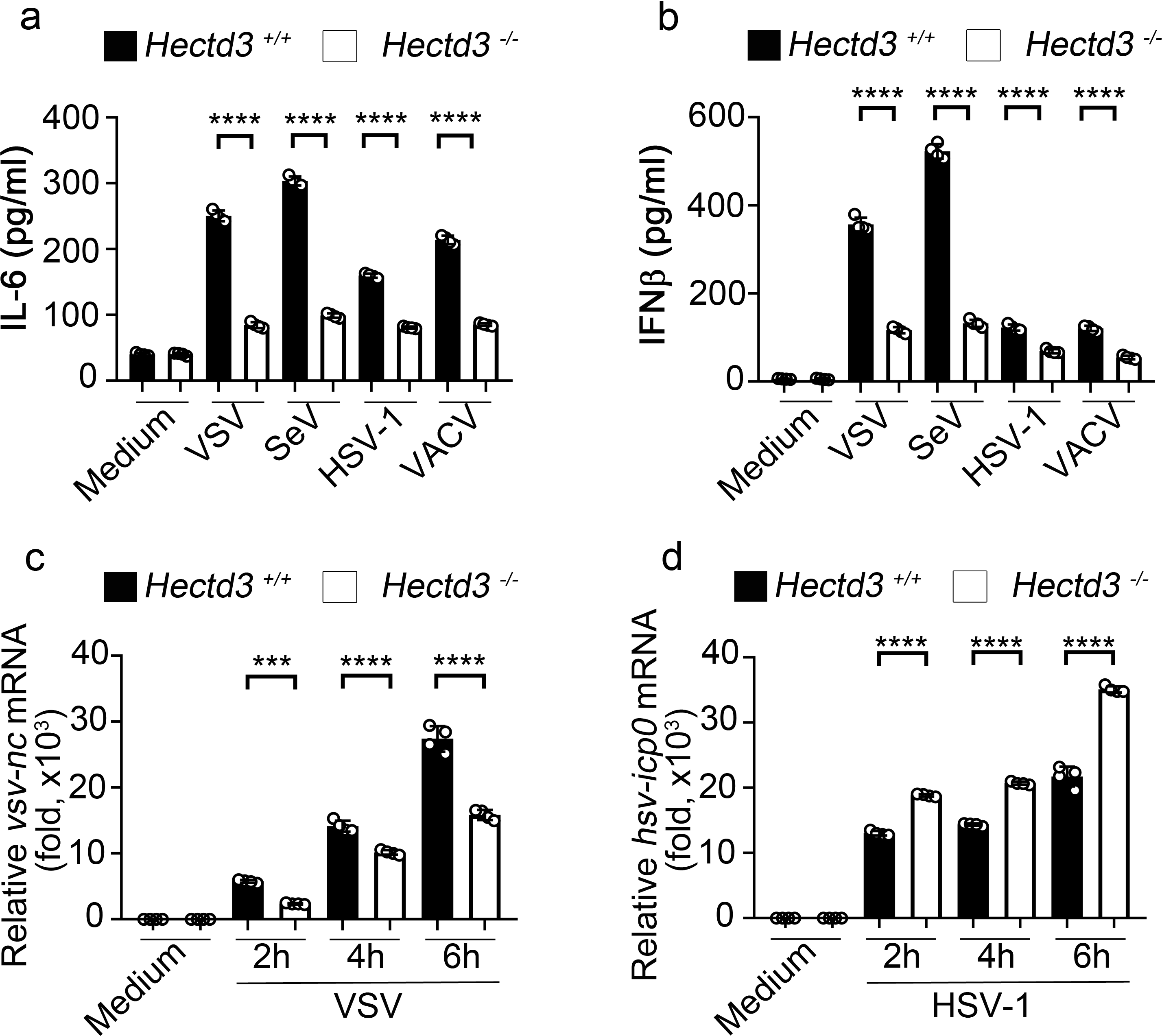


**Supplementary Fig.3 Knockout of *Hectd3* impairs innate immune response against VSV in L929 fibroblast cells.**

**a, b** *Hectd3*^+/+^ or *Hectd3*^–/–^ L929 fibroblast cells were infected with the indicated pathogens for 6h (MOI = 1 for VSV and SeV and MOI = 5 for HSV-1 and VACV). The amount of IL-6 (a) and IFNβ (b) in supernatants were measured by ELISA. **c, d** *Hectd3*^+/+^ or *Hectd3*^–/–^ L929 fibroblast cells were infected with VSV (MOI=1) (c) or HSV-1 (MOI=5) (d) for 2h, 4h, 6h. The mRNA level of VSV nucleocapsid (nc) (c) or HSV-1 ICP0 (d) gene was examined by Q-PCR. Results are presented as mean ± SD of triplicate samples (a-d; one-way ANOVA followed by Bonferroni multiple comparison). One representative data of three independent experiments are shown. ***, P < 0.001; ****, P < 0.0001.

**
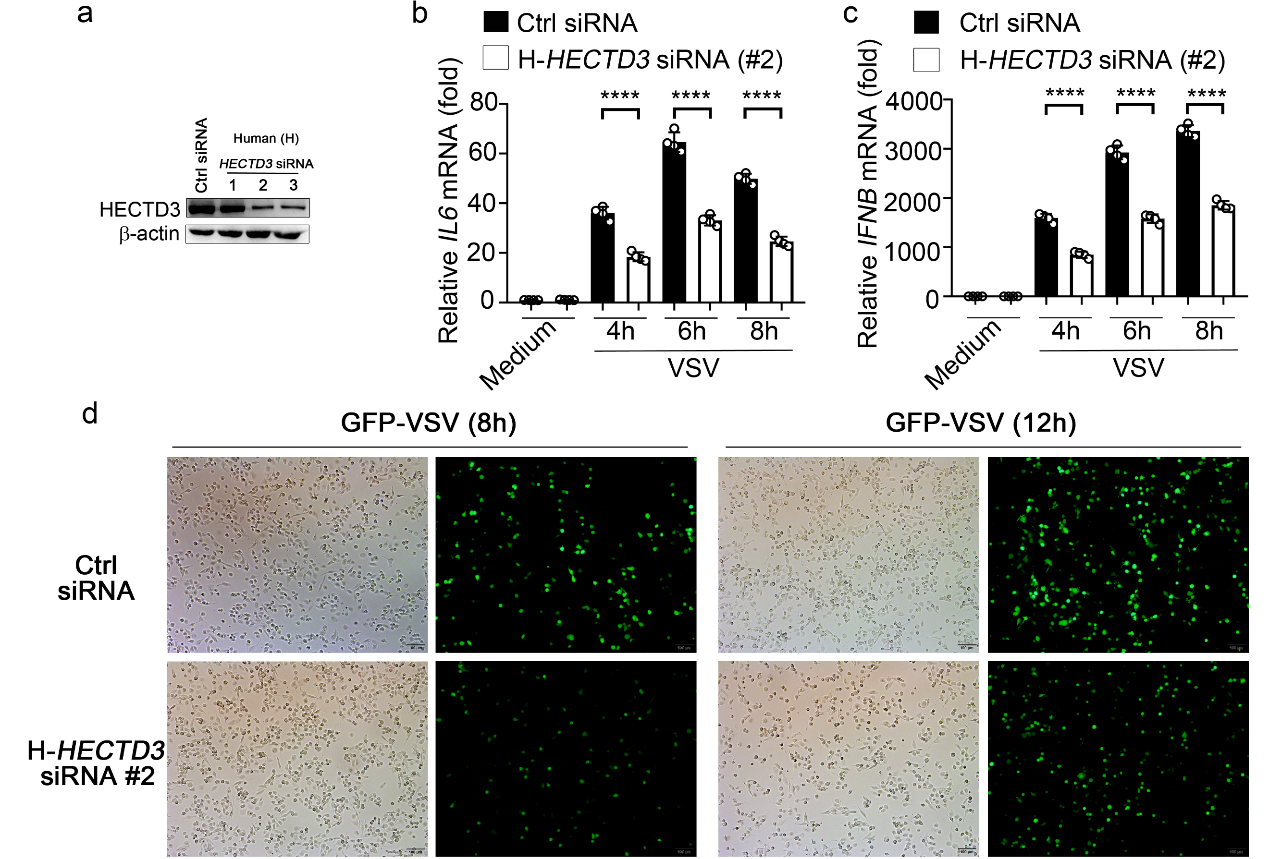
Supplementary Fig.4 Knockdown of HECTD3 impairs innate cytokine response to VSV and restrain virus replication in THP1 cells. a-c** THP1 cells were transfected with control (Ctrl) or human HECTD3-specific siRNAs for 48h. The efficiency of knockdown (KD) was evaluated by Western blotting (a). The cells were then infected with VSV (MOI = 1) as indicated. The mRNA levels of *IL6* (b) and *IFNB* (c) were evaluated by Q-PCR. **d** THP1 cells were transfected with control (Ctrl) or human HECTD3-specific siRNAs for 48h then infected with GFP-VSV (MOI=1) virus for 12h and examined by fluorescence microscope (Scale bar, 100 µm). Results are presented as mean ± SD of triplicate samples (b and c; one-way ANOVA followed by Bonferroni multiple comparison). One representative data of three independent experiments are shown. ****, *P*<0.0001.
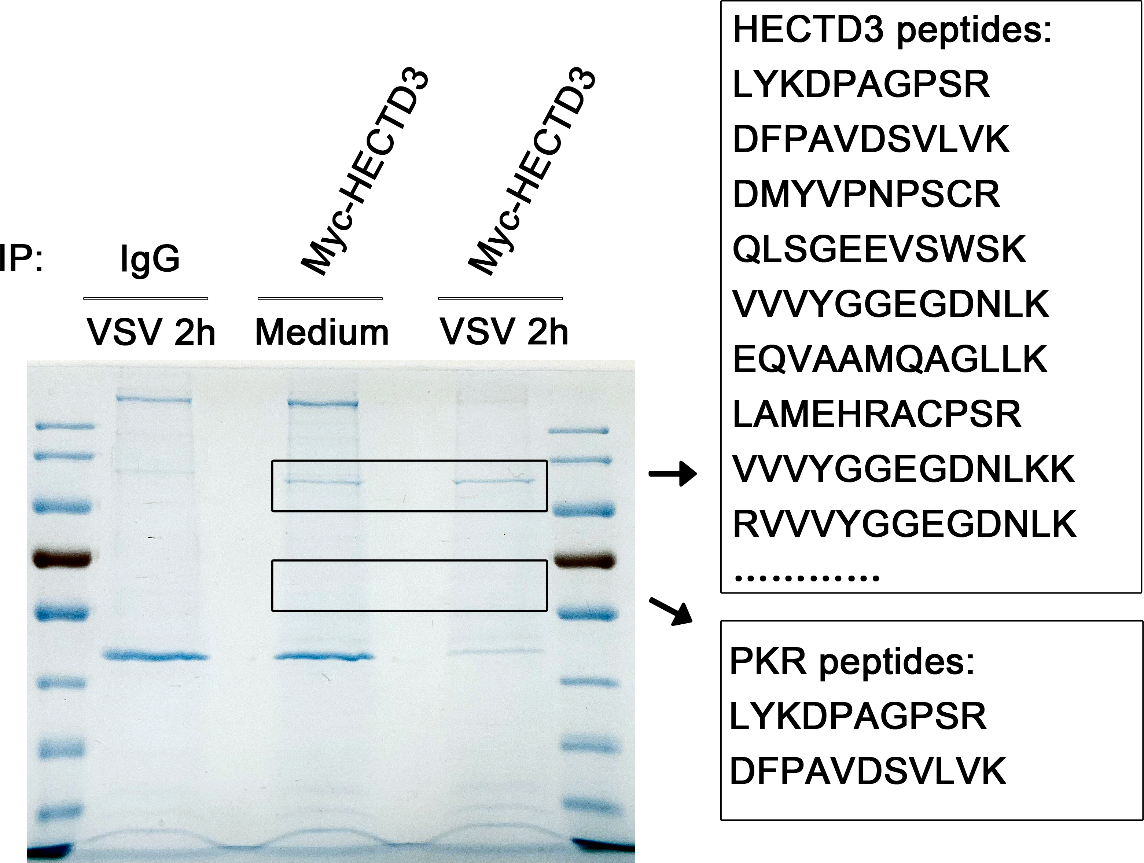


**Supplementary Fig.5 MS assays of HECTD3-associated proteins.** HEK293T cells were transfected with 5µg of a plasmid expressing Myc-HECTD3 for 48h then infected with VSV (MOI=1) as indicated. Whole cells lysates were immunoprecipitated (IP) with anti-Myc Sepharose Beads. The immune complexes were separated by SDS-PAGE and the gel was stained with Coomassie blue. Bands presented differently between lysates from infected and uninfected cells were subjected to MS assays. The identified unique peptides of HECTD3 and PKR are shown at the right side.

**
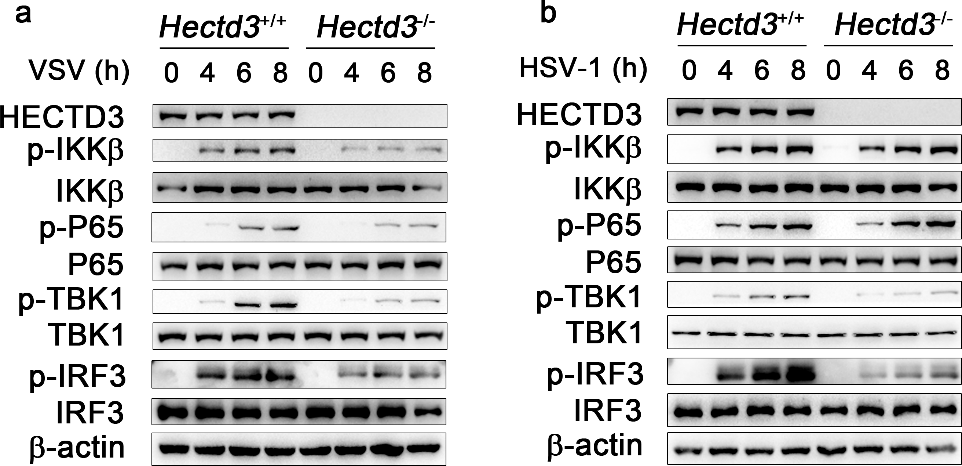
**

**Supplementary Fig.6 HECTD3 potentiates** **NF-κB and IRF3 activation** **in macrophages upon RNA virus infection. a, b** *Hectd3^+/+^* or *Hectd3^–/–^* BMDM were infected with VSV (MOI=1) (a) or HSV-1 (MOI=5) (b) for 0h, 4h, 6h, 8h as indicated and the activation of signaling mediators of NF-κB and IRF3 were examined by Western blotting.

**
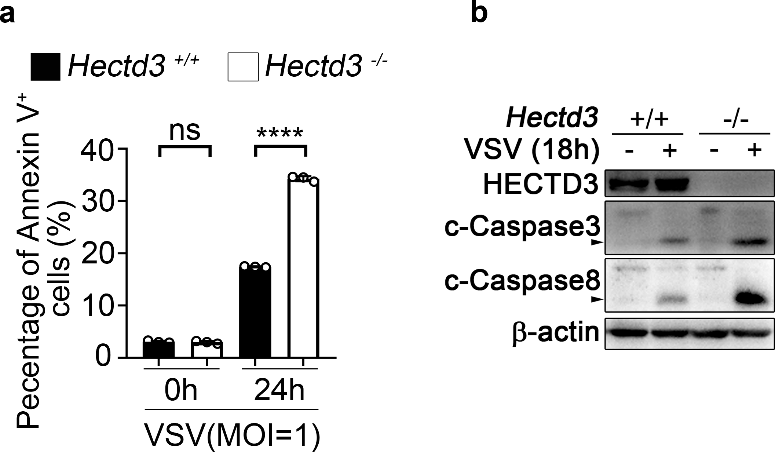
**

**Supplementary Fig.7 HECTD3 restrains VSV induced apoptosis of macrophages. a** Quantification of the percentage of ANNEXIN V^+^ *Hectd3^+/+^* or *Hectd3^–/–^* BMDM (2x10^5^ cells per 24-well) infected with VSV(MOI=1) for 24h as indicated. Cells were washed by PBS, stained with ANNEXIN V-FITC for 15 min at RT，and subjected to cell apoptosis analysis by flow cytometry. Results are presented as mean ± SD of three biological replicates (one-way ANOVA followed by Bonferroni multiple comparison). One representative experiment of three is shown. ns, no significance; ****, *P* < 0.0001. **b** *Hectd3^+/+^* or *Hectd3^–/–^* BMDM were infected with VSV (MOI=1) for 18h as indicated and the level of cleaved caspase3 and caspase8 were examined by Western blotting. One representative experiment of three is shown.

**
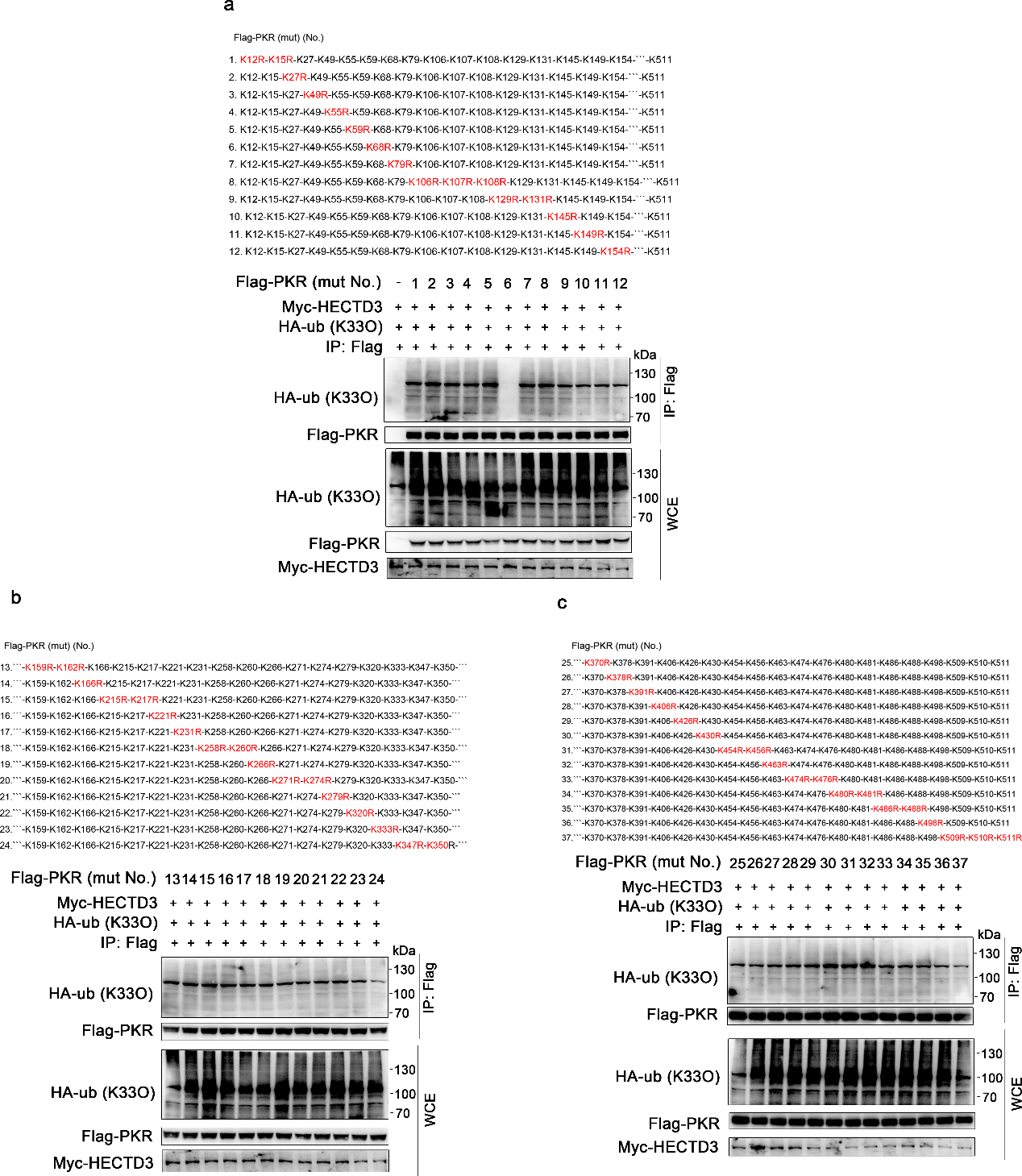
Supplementary Fig.8 Confirmation of the Lys residue in PKR responsible for linkage with K33O Ub chain.** **a-c** the mutants (1-37) were constructed as indicated. HEK293T cells were transiently transfected with indicated vectors for 48h and infected with VSV (MOI = 1) for 2h. Then Polyubiquitination of PKR (mutant) in immunoprecipitations (IPs) was evaluated by immunoblot (IB) (a, mut1-12; b, mut13-24; c, mut 25-37). One representative experiment of three was shown.

**
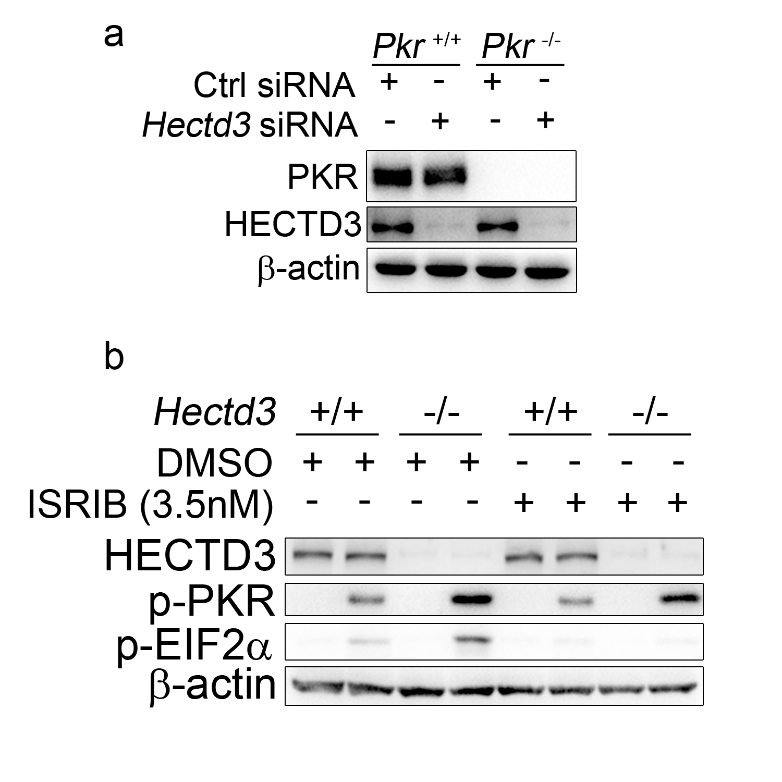
**

**Supplementary Fig.9 Knockdown of HECTD3 in *Pkr*^+/+^ and *Pkr*^-/-^ RAW264.7 cells and ISRIB inhibit the phosphorylation of EIF2α in BMDM. a** *Pkr*^+/+^ or *Pkr*^–/–^ Raw264.7 cells were transfected with control (Ctrl) or *Hectd3* siRNA (#2) as indicated. The efficiency of knockout and knockdown was evaluated by Western blotting. **b** *Hectd3*^+/+^ or *Hectd3*^–/–^ BMDMs were pretreated with DMSO or ISRIB (3.5nM) for 1h and infected with VSV (MOI=1) for 4h as indicated. The activation of the signaling mediators were examined by Western blotting.

**
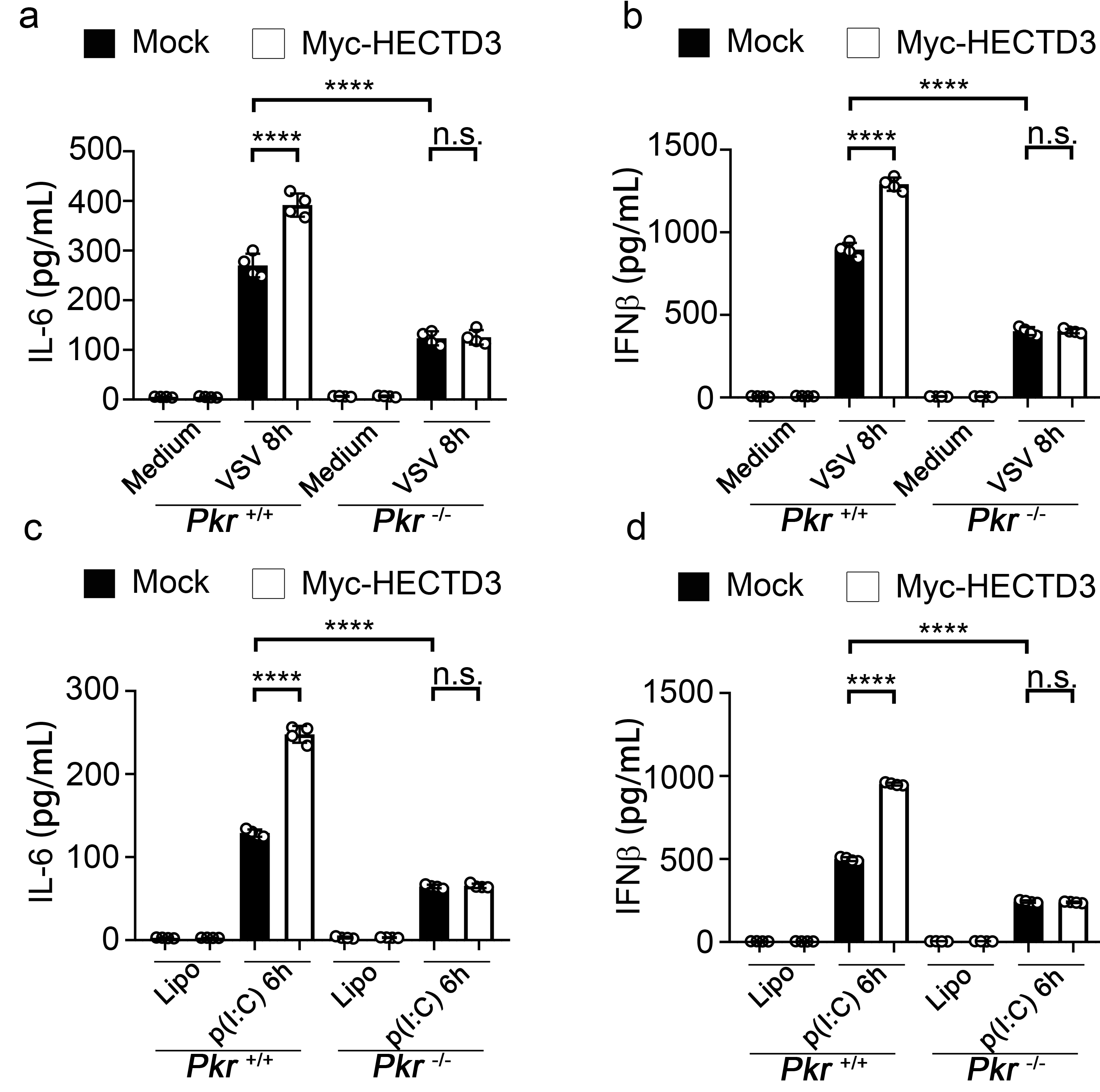
**

**Supplementary Fig.10 PKR is required for HECTD3-mediated proinflammatory cytokine expression. a-d** *Pkr^+/+^* or *Pkr^–/–^* Raw264.7 cells were transfected with a plasmid expressing Myc-HECTD3 and infected with VSV (MOI=1) (a, b) for 8h or transfected with poly(I:C) (c, d) for 6h as indicated. Amounts of IL-6 (a, c) and IFNβ (b, d) in supernatants were measured by ELISA**.** Results are presented as mean ± SD of triplicate samples (b and c; one-way ANOVA followed by Bonferroni multiple comparison). n.s., no significance; ****, P < 0.0001.

**Supplementary Table 1.** Sequences used in this study.

| Primers for genotyping of *Hectd3* knockout mice | | | |
| --- | --- | --- | --- |
| Primer name | Primer sequence (5’→3’) | Result judgment | |
| P1 | GACATATGAGTGCGACGAGGACCTG | P1+P2 (~550 bp, for 5’ arm)  Positive: Flox+  Negative: WT | |
| P2 | GACGTGCTACTTCCATTTGTCACG |  |  |
| P3 | CTATCGCCTTCTTGACGAGTTCTTC | P3+P4 (~700 bp, for 3’ arm)  Positive: Flox+  Negative: WT | |
| P4 | GGTATCTGCGTTGCTATCTGTCAG |  |  |
| P5 | GCGCTGGAGTTTCAATACCG | P5+P6 (~325 bp)  Positive: EIIA-Cre+  Negative: EIIA-Cre ^−^ | |
| P6 | TTTGACAGGAAACGCAACGG |  |  |
| P7 | CTTCATGGTAAGCCGTACCCAGTG | P7+P8 (~550 bp)  Positive: Hectd3^−/−^ or Hectd3^+/−^  Negative: Hectd3^+/+^ | |
| P8 | CACCTGAGTGACTGTAGTCTGGACG |  |  |
| P9 | GGAACCACTTAGGAAGCATCTAG | P8+P9 (~425 bp)  Positive: Hectd3^+/+^ or Hectd3^+/−^  Negative: Hectd3^−/−^ | |
|  |  |  |  |
| *Hectd3*-specific targeted sequences for CRISPR-Cas9 edition | | | |
| Sequence No. | Sequence | KO efficiency | |
| 1 | GACAGTATCGAGCTCCGGCG | No | |
| 2 | GCAAGCCCAGCTTCATGCCG | No | |
| 3 | GGGTAGTGTGAAGCAGTATG | Yes | |
| 4 | CAGCCAGCAGAGGCATATAC | No | |
| 5 | GCTTCATGCCGAGGCTGTAC | Yes | |
| *Pkr*-specific targeted sequences for CRISPR-Cas9 edition | | | |
| Sequence No. | Sequence | KO efficiency | |
| 1 | TATAAAGAACTTAGTACTT | Yes | |
| 2 | CAAAGAAATAAATATACCT | Yes | |
| 3 | AGGATCGGCAAGCTTTAG | Yes | |
| 4 | AACATCCTCTAGCGTTGTC | No | |
| *Hectd3-homo-*specific siRNA sequences | | | |
| Sequence name | Sequence (5’→3’) | Knockdown efficiency | |
| siRNA1 | GGACCUGACAUACUCACAUTT | High | |
|  | AUGUGAGUAUGUCAGGUCCTT |  |  |
| siRNA2 | GGCACCUUCAGUGAGAUUATT | Highest and selected | |
|  | UAAUCUCACUGAAGGUGCCTT |  |  |
| siRNA3 | CCGAGACUUUGCCAAGUAUTT | High | |
|  | AUACUUGGCAAAGUCUCGGTT |  |  |
| *Hectd3-mus-*specific siRNA sequences | | | |
| Sequence name | Sequence (5’→3’) | | Knockdown efficiency |
| siRNA1 | ACACCUGAGUGACUGUAGUTT | | High |
|  | ACUACAGUCACUCAGGUGUTT | |  |
| siRNA2 | CCUAUACGGAGGAGUUCAATT | | High and selected |
|  | UUGAACUCCUCCGUAUAGGTT | |  |
| siRNA3 | GCACCUUUAGUGAGAUUAATT | | High |
|  | UUAAUCUCACUAAAGGUGCTT | |  |
| Primers for Q-PCR assays | | | |
| Gene name | Primer sequence (5’→3’) | | |
|  | Sense | Anti-sense | |
| Mouse *Il6* | GAGTTGTGCAATGGCAATTCTG | GCAAGTGCATCATCGTTGTTCAT | |
| Mouse *Ifnb* | CAGCTCCAAGAAAGGACGAAC | GGCAGTGTAACTCTTCTGCAT | |
| Mouse *Actb* | AGTGTGACGTTGACATCCGT | GCAGCTCAGTAACAGTCCGC | |
| Human *Il6* | ACTCACCTCTTCAGAACGAATTG | CCATCTTTGGAAGGTTCAGGTTG | |
| Human *Ifnb* | GTCACTGTGCCTGGACCATAG | GTTTCGGAGGTAACCTGTAAGTC | |
| Human *Actb* | CATGTACGTTGCTATCCAGGC | CTCCTTAATGTCACGCACGAT | |
| VSV NC | ACGGCGTACTTCCAGATGG | CTCGGTTCAAGATCCAGGT | |
| HSV-1 ICP0 | GATGCAATTGCGCAACAC | GCGTCACGCCCACTATCAG | |

**Supplementary Table 2.** Antibodies used in this study.

| **Company** | **Product name** | **Clone No.** | **Catalog No.** |
| --- | --- | --- | --- |
| Cell Signaling Technology (Beverly, MA) | Anti- phospho-p65(Ser536) | 93H1 | 3033 |
|  | Anti- IKKβ | D30C6 | 8943 |
|  | Anti-Flag tag | 9A3 | 8146 |
|  | Anti-HA tag | C29F4 | 3724 |
|  | Anti-IRF3 | D83B9 | 4302 |
|  | Anti-Myc tag | 71D10 | 2278 |
|  | Anti-p65 | L8F6 | 6956 |
|  | Anti-phospho-IRF3 (Ser396) | 4D4G | 4947 |
|  | Anti-phospho-TBK1 (Ser172) | D52C2 | 5483 |
|  | Anti-TBK1 | D1B4 | 3504 |
|  | Anti-cleaved caspase8(Asp387) | NA | 9429 |
|  | Anti-cleaved caspase3 | 5A1E | 9664 |
|  | Anti-Ubiquitin | P4D1 | 14049 |
|  | Anti-K48 specific polyubiquitin | D9D5 | 8081 |
|  | Anti-K63 specific polyubiquitin | D7A11 | 5621 |
|  | Anti-TRAF3 | E8H3B | 36640 |
|  | Anti-phospho-eIF2alpha (S51) | D9G6 | 3398 |
|  | Anti-eIF2alpha | D7D3 | 5324 |
|  | Normal rabbit IgG | NA | 2729 |
| Medical&biological laboratories co.,LTD | Anti-IKKγ | NA | K0159 |
| Abcam Inc. (Cambridge, MA) | Anti-HECTD3 | NA | Ab241413 |
|  | Anti-Flag-tag | M2 | ab49763 |
|  | Anti-HA-tag | HA.C5 | ab18181 |
|  | Anti-Myc-tag | Myc.A7 | ab18185 |
|  | Anti-PKR | NA | ab184257 |
|  | Anti-phospho-PKR(T446) | NA | Ab32036 |
| Servicebio (Wuhan, CHN) | Anti-CD45 | NA | GB11066 |
|  | Cy3 conjugated Goat Anti-Rabbit IgG (H+L) | NA | GB21303 |
| Novus | K33 linkage-polyubiquitin antibody | NA | NBP3-05656 |
| BD Biosciences | BD Pharmingen™ FITC Rat Anti-Mouse CD45 | 30-F11 | 553079 |
|  | BD Pharmingen™ APC Mouse Anti-Mouse NK-1.1 | PK136 | 550627 |
|  | BD Pharmingen™ FITC Rat Anti-Mouse CD4 | GK1.5 | 553729 |
|  | BD Pharmingen™ PE Rat Anti-Mouse CD8a | 53-6.7 | 553033 |
|  | BD Pharmingen™ APC Rat Anti-Mouse CD3 Molecular Complex | 17A2 | 565643 |
|  | BD Pharmingen™ PE Rat Anti-Mouse CD19 | 1D3 | 553786 |
|  | BD Pharmingen™ PE Hamster Anti-Mouse CD11c | HL3 | 553802 |
|  | BD Pharmingen™ FITC Mouse Anti-Mouse I-A[b] | AF6-120.1 | 553551 |
|  | BD Pharmingen™ FITC Rat Anti-CD11b | M1/70 | 553301 |
|  | BD Pharmingen™ APC Rat Anti-Mouse Ly-6G | 1A8 | 560599 |
|  | BD Pharmingen™ PE Rat Anti-Mouse F4/80 | T45-2342 | 565410 |

NA, not available.

|  |
| --- |
